# Supplementary material for: Formation of spatial vegetation patterns in heterogeneous environments
Source: PLoS One. 2025 May 28;20(5):e0324181. doi: 10.1371/journal.pone.0324181 (PMC12118857; doi:10.1371/journal.pone.0324181)
Supplement: S1 Appendix — (PDF) [file pone.0324181.s001.pdf]

Supplement of: Formation of spatial vegetation patterns in heterogeneous environments

Karl Kästner[1], Daniel Caviedes-Voullième[3,4], and Christoph Hinz[1]

[1]Hydrology, BTU Cottbus-Senftenberg, 03046 Cottbus, Germany
[3]Institute of Bio- and Geosciences: Agrosphere (IGB-3), Forschunszentrum Jülich, 52428 Jülich, Germany
[4]Simulation and Data Lab Terrestrial Systems, Jülich Supercomputing Centre (JSC), 52425 Jülich, Germany

March 30, 2025

In this supplement, we elaborate the details of our analysis which has been kept brief in the main manuscript.

Contents

List of symbols **3**

1 Pattern formation as a filtration **4**
1.1 Linearization 5
1.2 Space-invariant approximation 6
1.3 Filtering in the spatial domain 7
1.4 Filtering in the spectral domain 8
1.5 Estimating linear space-invariant filters 8
1.6 Thresholding 10
1.7 Modelling the heterogeneity 10
1.7.1 Discretization of the heterogeneity 11

2 Parametric linear filters **13**
2.1 Irregular patterns 16
2.1.1 A nonlinear reaction-diffusion model for irregular patterns . . 16
2.1.2 Irregular pattern formation by low-pass filtering 18
2.2 Regular patterns 20
2.3 Isotropic regular patterns 20
2.3.1 Linear filters for isotropic patterns 20
2.3.2 Band-pass filter 21
2.4 Anisotropic regular patterns 22
2.4.1 Linear filter for regular anisotropic patterns 22

2.4.2 Linear oscillator with phase noise integration 23
2.4.3 Nonlinear oscillator with phase noise integration 24

3 Fitting parametric densities and predicting patterns **25**
3.1 Predicting patterns from 27
3.2 Goodness of fit of patterns predicted by linear filtration 27
3.3 Random phase 28

e

List of symbols

We follow the methodology and notation for spectral and spatial analysis as detailed in *Kästner et al.* (2024a) and *Kästner et al.* (2024b).

For operators, calligraphic font indicates the continuous, infinite dimensional form, and normal font their discretized forms.

Symbols used for the Rietkerk and May model are tabulated at introduction of the respective model.

a	1) coefficients of the linearized reaction part
	2) infiltration coefficient of the RK-model
$\mathcal{A}$	differential operator combining reaction, diffusion and advection
$\bar{e}$	mean over the spatially heterogeneous parameter
b	z_1 (biomass)
$\mathcal{D}$	difference operator
d	diffusion coefficient
Δt	finite time step
det	determinant, for a 2x2 operator, $det(A) = a_{11}\, a_{22} - a_{12}\, a_{21}$
e	spatially heterogeneous parameter
$\mathcal{F}$	Fourier transform
Γ	Gamma function
$\mathcal{I}$	identity operator
$\imath$	imaginary number
$\mathcal{IR}$	impulse response
J	Bessel function
k_c	characteristic wavenumber, wave number at the maximum of the spectral density
k	wave number (when not used as index)
λ	wavelength
λ_c	characteristic wavelength
$\mathcal{L}$	linear operator $\mathcal{L}^{-1} = (\frac{\partial \mathcal{A}}{\partial z})^{-1} \frac{\partial \mathcal{A}}{\partial e}$
L	side length of a square domain
φ phase	
p	1) fraction of ground covered by vegetation
	2) parameter of linear filters
R^2	goodness of fit
$\mathcal{R}$	autocorrelation function
r	radial coordinate
ρ	one-tap correlation of the discrete low-pass filter (AR1 process)
$\mathcal{S}_c$	maximum of the spectral density
σ^2	variance
$\mathcal{S}$	spectral density
S	discretized spectral density
$\hat{S}$	periodogram

θ	angular coordinate
t	time
$\mathcal{T}$	transfer function
v	velocity of surface water in the Rietkerk model
w	1) weight for regression and averaging
	2) soil water content
x	spatial coordinate (chosen to be perpendicular to stripes)
y	spatial coordinate (chosen to be parallel to stripes)
z_*	spatially homogeneous state at which system is linearized
z_∞	stationary state
$z_\mathcal{L}$	state of the linearized system
z	system state b,w,h
$\cdot$	point wise multiplication
$\div$	point wise wise division
$\circledast$	convolution

1 Pattern formation as a filtration

We consider a pattern forming ecosystem which can be modelled continuously in time and space, i.e. by considering the system state, such as plants as averaged over individuals into a local biomass concentration and the influence of events such as precipitation as averaged over time. The evolution of the pattern z over time of such as system can described the nonlinear partial differential equation

$$\frac{\partial z}{\partial t} = \mathcal{A}(z, e). \tag{SI 1}$$

Where $\mathcal{A}$ is the differential operators determining how the pattern evolves, e is an exogenous random spatial heterogeneity, such as varying soil properties. The system state $z(t, \vec{x})$ consists at least of the biomass concentration b and quantities it interacts with that are relevant for the pattern formation. For the grazing model (*May*, 1977), $z = b$, and for the Rietkerk model (*Rietkerk et al.*, 2002) $z = [b, w, h]^T$, with soil moisture w, and surface water h.

When the environmental conditions do not change over time, i.e. when the operators do not explicitly depend on time, and the heterogeneity is only spatial, then the system is autonomous, and can approach a stationary state z_∞, where the pattern does not change over time anymore $\frac{\partial z}{\partial t} = 0$:

$$0 = \mathcal{A}(z_\infty, e). \tag{SI 2}$$

$\mathcal{A}$ can be interpreted as a filter, which amplifies and suppresses components of the spatial heterogeneity e depending on their wavelength or corresponding wavenumber, and generates new components by the nonlinear interaction of the components. $\mathcal{A}$ therefore transforms the exogenous heterogeneity e into the pattern z_∞.

The stationary state z_∞ is not unique, as it also depends on the initial and boundary conditions. Furthermore, when the heterogeneity is random every spatial heterogeneity map results in a different pattern. This is also what we observe in nature,

where no two regular patterns are identical. However, patterns from the same region appear still similar, i.e. they have a similar spatial structure, in particular a similar regularity. This is because the exogenous heterogeneity determines the spatial structure of the pattern. We therefore focus on the influence of the spatial heterogeneity of e on the pattern here.

Some systems do not reach a stationary state. This is in particular the case in anisotropic systems, for example for arid ecosystems forming vegetation stripes on hillslopes, which perpetually migrate uphill and respawn at the downstream end. In rare cases, isotropic systems cycle as well (*Johnston and Greene*, 2022). However, cyclic systems can still reach a state where spectral density $\mathcal{S} = |\mathcal{F}(z)|^2$ which is stationary in time:

$$\frac{\partial \mathcal{S}}{\partial t} = 0. \tag{SI 3}$$

i.e. the average magnitude of the frequency components remains constant and only the phase varies with time. The system then still can be interpreted as a filter which amplifies and suppresses the magnitude of the frequency components of the heterogeneity. While pattern forming ecosystems are typically nonlinear their response to exogenous heterogeneity can be approximated by linearization, which we elaborate on in the remainder of the section.

1.1 Linearization

When $\mathcal{A}$ is continuously differentiable then the evolution of the system in time close to any state z_* with spatially varying parameter e can be approximated by series expansion. Considering only constant and linear terms, i.e. neglecting quadratic and higher terms the series expansion is:

$$\frac{\partial (z_l - z_*)}{\partial t} = \mathcal{A}_* + \frac{\partial \mathcal{A}}{\partial z}(z_l - z_*) + \frac{\partial \mathcal{A}}{\partial e}(e - e_*). \tag{SI 4}$$

Where z_l is the state of the linear system, $\mathcal{A}_* = \mathcal{A}(z_*, e_*)$ the rate of change and $\frac{\partial \mathcal{A}}{\partial z}$, $\frac{\partial \mathcal{A}}{\partial e}$ the Jacobians at the state z_* spatially heterogeneous parameter e_*.

The pattern of the linear system with initial state $z_l(0) = z_0$ evolves over time as:

$$z_l(t) = z_{l*} - \frac{\partial \mathcal{A}}{\partial z}^{-1} \left(\mathcal{I} - \exp\left(t\frac{\partial \mathcal{A}}{\partial z}\right)\right) \left(\mathcal{A}_* + \frac{\partial \mathcal{A}}{\partial e}(e - e_*)\right) + \exp\left(t\frac{\partial \mathcal{A}}{\partial z}\right)(z_0 - z_*). \tag{SI 5}$$

Where exp is the operator, i.e. matrix, exponential.

When the operator $\frac{\partial \mathcal{A}}{\partial z}$ is stable, i.e. when the real parts of all its eigenvalues are negative, then then linear system approaches the stationary state $z_{l\infty}$:

$$z_{l\infty} = z_* - \left(\frac{\partial \mathcal{A}}{\partial z}\right)^{-1} \left(\mathcal{A}(z_*, e_*) + \frac{\partial \mathcal{A}}{\partial e}(e - e_*)\right). \tag{SI 6}$$

The spatial variation of the linearized system z_l is thus uniquely determined by the exogenous spatial heterogeneity e.

As the pattern fluctuates between a low and high state, the distance to the state at which the pattern is linearized is small ($|z - z*| < 1/2|z_{\max} - z_{\min})$ when the system is linearized close to the midpoint. The linearization therefore remains valid within the parameter range of possible states and the magnitude, and hence influence, of the nonlinear higher order terms therefore decreases exponentially.
The system responds to a local perturbation differently depending on if the ground is vegetated or bare. The linear operator $-\left(\frac{\partial\mathcal{A}}{\partial z}\right)^{-1}\mathcal{A}(z_*, e_*)$ therefore varies in space depending on the particular spatial map e, when the system is linearized in a particular patterned state.

1.2 Space-invariant approximation

The spectral response of a system, i.e. $\mathcal{S}_\infty = |F(z_\infty)|^2$, can be determined by linearizing it in a homogeneous state, as long as the statistical properties of the exogenous heterogeneity do not change within the spatial extent of a pattern. This is because the spatial structure remains constant throughout the pattern, i.e. the pattern can be tessellated into tiles with identical spectral density and autocorrelation. All tiles respond statistically in the same way to perturbations.

When the system is linearized in a spatially homogeneous state $z_*, e_* = \bar{e}$: then the linear operator is spatially homogeneous, i.e. space-invariant, as well. Collecting the spatial mean in the variable $\bar{z}_l = z_* + \left(\frac{\partial\mathcal{A}}{\partial z}\right)^{-1}\mathcal{A}(z_*, \bar{e})$, the stationary state is:

$$z_{l\infty} = \bar{z}_l - \left(\frac{\partial\mathcal{A}}{\partial z}\right)^{-1}\frac{\partial\mathcal{A}}{\partial e}(e - \bar{e}) \tag{SI 7}$$

The linear operator $\frac{\partial\mathcal{A}}{\partial z}^{-1}\frac{\partial\mathcal{A}}{\partial e}$ can be interpreted as a filter which, in accordance to its eigenfunctions and eigenvalues, amplifies and suppresses spatial components of the exogenous heterogeneity e depending on their wavelength. The homogeneous operator $\left(\frac{\partial\mathcal{A}}{\partial z}\right)^{-1}\frac{\partial\mathcal{A}}{\partial e}$ does predict the equilibrium pattern $z_{l\infty}$ for any spatial heterogeneity map e with the same statistical properties, i.e. same variance and correlation structure. The linear operator therefore determines the spatial structure of the pattern, in particular its regularity. The linear operator has to be re-estimated for different environmental conditions, such as a different noise magnitude, or different water availability.

A linear space-invariant approximation of a reaction-advection-diffusion model has typically the form of a block-matrix:

$$\frac{\partial\mathcal{A}}{\partial z} = \begin{pmatrix} \frac{\partial\mathcal{A}}{\partial z}_{11} & \cdot & \frac{\partial\mathcal{A}}{\partial z}_{1k} \\ \cdot & \cdot & \cdot \\ \frac{\partial\mathcal{A}}{\partial z}_{k1} & \cdot & \frac{\partial\mathcal{A}}{\partial z}_{kk} \end{pmatrix}, \tag{SI 8a}$$

$$\frac{\partial A}{\partial e} = \begin{pmatrix} \frac{\partial A}{\partial e}_1 \\ \cdots \\ \frac{\partial A}{\partial e}_k \end{pmatrix}. \tag{SI 8b}$$

Where z consists of the components $z = (z_1, \ldots, z_k)$, where z_i is the two-dimensional spatial map of each state variable. For the Rietkerk model these are biomass $z_1 = b$, soil water $z_2 = w$ and surface water $z_3 = h$.
The linearized spatially homogeneous operator has the structure:

$$\frac{\partial \mathcal{A}}{\partial z}_{ii} = a_{ii}\mathcal{I} + v_{xi}\mathcal{D}_x + v_{yi}\mathcal{D}_y + d_{xi}\mathcal{D}_x^2 + d_{yi}\mathcal{D}_y^2, \tag{SI 9a}$$

$$\frac{\partial \mathcal{A}}{\partial z}_{ij} = a_{ij}\mathcal{I}, \quad i \neq j. \tag{SI 9b}$$

Where $\mathcal{I}$ is the identity operator and $\mathcal{D}$ the derivative operator. a_{ij} are the reaction coefficients and d_i the diffusion coefficients and v the velocity. When parameters of the reaction part are spatial heterogeneous, then $\frac{\partial A}{\partial e}$ does not contain derivative operators and therefore simplifies to a scalar in the homogeneous case.

As the operator is space-invariant, the rows of the discretized operator are identical, up to a shift in position.This facilitates filtering in the spectral domain, as it is only necessary to evaluate the filter row with n^2 elements once, instead of evaluating n^2 rows with each n^2 elements. Vice versa, it facilitates estimating the filter from the frequency spectrum of a pattern.

The response of the linear system is highly sensitive to the state z_* where the nonlinear system is linearized. For most states, the linearized system responds quite differently to the nonlinear system, i.e. $||z_{l\infty} - z_\infty||_2$ is large. This is also the case for stationary homogeneous states, i.e. critical points, at which deterministic systems are typically linearized. To predict the response of the nonlinear system reliably, it has to be linearized statistically, i.e. by averaging the multiple states z_* and their corresponding linear operators $\frac{\partial A}{\partial z}$. As the homogeneous linear operator has a finite number of parameters, it is possible to find a linear operator which is optimal in the least square sense as a weighted average of a finite number of states. As the number of parameters in the linearized system is small, it is also possible to fit them directly, instead of determining them by averaging homogeneous states. In the following section, we show that it is possible to find a linear operator just from a spatial map or orthographic image of a pattern without any additional information on the system itself. In section 2 we introduce simplified parametric forms for the linearized operators, which have just two parameters for regular isotropic patterns.

1.3 Filtering in the spatial domain

In the remainder of the section, we focus on the stationary state of the linearized system and for ease of notation drop the (l, ∞) from the indices. The stationary state of the i-th state variable z_i of the linearized system is:

$$z_i = \bar{z}_i + \sum_{j=1}^{k} \left(\left(\frac{\partial \mathcal{A}}{\partial z} \right)^{-1} \right)_{ij} \left(\frac{\partial \mathcal{A}}{\partial e} \right)_j e, \tag{SI 10a}$$

$$= \bar{z}_i + \mathcal{L}_i^{-1} e, \tag{SI 10b}$$

where $\mathcal{L}_i^{-1}$ is the combined linear operator.

As the system is linear and spatially homogeneous, the rows of the blocks of $\left(\frac{\partial \mathcal{A}}{\partial z}^{-1}\right)_{ij}$ are identical up to a shift in location and correspond to the impulse response $\mathcal{IR}_{ij}$ of the block. The filtering can therefore be conducted by convolution:

$$z_i = \bar{z}_i + \sum_{j=1}^{k} \mathcal{IR}_{ij} \circledast \left(\left(\frac{\partial \mathcal{A}}{\partial e} \right)_j e \right) \quad \text{(SI 11a)}$$

$$= \bar{z}_i + \mathcal{IR}_i \circledast e \quad \text{(SI 11b)}$$

Where $\mathcal{IR}_i$ is the impulse response of the combined operator $\mathcal{L}_i^{-1}$. The impulse response of a linear filter is identical to filtering the Dirac pulse. This can be approximated in a discrete system by filtering a spatial map that has the value $(\Delta x \Delta y)^{-1}$ at the origin and zero elsewhere.

1.4 Filtering in the spectral domain

The heterogeneity can be conversely filtered in the frequency domain, by multiplying its Fourier transform with the transfer function $\mathcal{T}$ of the linear system:

$$z_i = \bar{z}_i + \sum_{j=1}^{k} \mathcal{F}^{-1} \left(\mathcal{T}_{ij} \cdot \mathcal{F} \left(\left(\frac{\partial \mathcal{A}}{\partial e} \right)_j e \right) \right) \quad \text{(SI 12a)}$$

$$= \bar{z}_i + \mathcal{F}^{-1}(\mathcal{T}_i \cdot \mathcal{F}(e)) \quad \text{(SI 12b)}$$

Where $\mathcal{T}_{ij}$ is the transfer function of the blocks of the operator $\frac{\partial \mathcal{A}}{\partial z}^{-1}$ and $\cdot$ the point-wise product, and $\mathcal{T}_i$ the transfer function of the combined operator $\mathcal{L}_i^{-1}$.

The spectral density $\mathcal{S}_i = |\mathcal{F}(z_i)|^2$ of the spatial pattern of the i-th state variable is therefore:

$$\mathcal{S}_{z_i} = s\, \mathcal{S}_i \cdot \mathcal{S}_e. \quad \text{(SI 13)}$$

Where $\mathcal{S}_i = |\mathcal{T}_i|^2$ is the spectral density of the operator $\mathcal{L}_i$ and $\mathcal{S}_e = |\mathcal{F}(e)|^2$ is the spectral density of the spatial heterogeneity, and s a scale factor so that the volume of $\mathcal{S}_{z_i}$ integrates to 1.

1.5 Estimating linear space-invariant filters

The linear filter can be estimated from the spatial map of a state variable of a pattern, such as an orthographic image of the vegetation. Images are typically discretized on a square uniform grid so that each z_i has $n_x \times n_y$ elements. This discretizes both in the spatial and spectral domain with $\Delta x = L/n$ and $\Delta k = 2\pi/L$. We distinguish the continuous from the discrete operators, by writing the former in cursive and the letter in roman font.

As the linear operator can be decomposed into components, the linear filter of each state variable can be estimated independently of the other state variables, i.e. the filter for the biomass can be estimated from an orthographic image of the biomass only, without any information on the water distribution. The periodogram

$\hat{S}_{z_i} = |\mathcal{F}(z_i)|^2$ of the pattern sampled over a finite spatial extent with finial spatial resolution is, c.f. equation SI 13:

$$\sigma^2_{z_i}\,\hat{S}_{z_i} = (\sigma^2_{L_i}\,S_{L_i}) \cdot (\sigma^2_e\,\hat{S}_e) \tag{SI 14}$$

Where S_{L_i} is the spectral density of the discrete filter L_i, $\sigma^2_{z_i}$ and σ^2_e are the spatial variances of the pattern and the exogenous heterogeneity, and $\sigma^2_{L_i}$ a frequency independent scale of variance.

$$S_{L_i} = \frac{\sigma^2_{z_i}}{\sigma^2_e \sigma^2_{L_i}} (\hat{S}_{z_i} \div \hat{S}_e) \tag{SI 15}$$

Which is a deconvolution of the pattern and the noise. For a natural pattern, the spatial map of the heterogeneity and therefore its periodogram $\hat{S}_e$ is not known. However, we can assume a particular form of the noise spectrum $S_e = E[\hat{S}_e]$. The spectral density of the pattern $S_{z_i} = E[\hat{S}_{z_i}]$ can be consistently estimated as $\bar{S}_{z_i}$, either by smoothing the periodogram of a stationary pattern $\hat{S}_{z_i}$, or by fitting a parametric density:

$$S_{L_i} = \frac{\sigma^2_{z_i}}{\sigma^2_e \sigma^2_c} (\bar{S}_{z_i} \div S_e) \tag{SI 16}$$

Where $\div$ denotes pointwise division. In the particular case that the noise e is uncorrelated, i.e. white, then S_e is flat so that:

$$S_{L_i} = s^2\,\bar{S}_{z_i} \tag{SI 17}$$

Where $s^2 = \frac{\sigma^2_{z_i}}{\sigma^2_e \sigma^2_c}$ is a scalar constant determining the rate at which the pattern reaches a stationary state, which can be set an to arbitrary value if one is only interested in the equilibrium pattern.

In isotropic patterns, patches are neither oriented nor aligned in a particular direction. The impulse response is therefore symmetric, and the transfer function T is real with positive values, and can be determined as the square root of the spectral density:

$$T_i = s\sqrt{\bar{S}_i}. \tag{SI 18}$$

Where T_i is the discrete transfer function with $n_x \times n_y$ elements. The impulse response IR of the discretized operator L_i^{-1} is the inverse Fourier transform:

$$IR_i = \mathcal{F}^{-1}(T_i) \tag{SI 19}$$

and has also $n_x \times n_y$ elements. The rows of the discretized linear operator L_i^{-1} are identical to the impulse response IR_i flattened to a vector and shifted in space.

We can therefore estimate a homogeneous linear filter from an orthographic image of a pattern and a reasonable assumption about the spectrum of the exogenous heterogeneity. This filter will generate patterns with similar spatial structure as

the original pattern for which the filter is estimated. Furthermore, given the spatial map of the heterogeneity under which the original pattern formed, the filter can approximate the original pattern. As the model is linear the phase of the frequency components of the predicted pattern is identical to that of the corresponding frequency components in the heterogeneity. The phase of the linearly predicted frequency components are thus linearly independent and random, as they are linearly independent and random in the heterogeneity. The linear filter does not predict the frequency components introduced by the nonlinearity. These frequency components are mutually dependent and associated with the sharp transition from bare ground to vegetated patches, and asymmetry of striped vegetation patterns, which typically have a higher biomass concentration at their uphill side than at their downhill side.

1.6 Thresholding

The linear filter does not predict the bimodality of the biomass distribution, as this is determined by the phase relation between the frequency components. The linear filter consequently cannot distinguish between spotted, labyrinthine or gapped patterns. However, a pattern predicted by the linear filter can be simply transformed into a pattern with bimodal biomass distribution, for example by thresholding. Spotted, labyrinthine and gapped patterns emerge from the same unimodal pattern for different levels of thresholding.

For the Rietkerk model, for example, the mean biomass and fraction of the ground covered by vegetation depend nearly linearly on water availability as long as the water availability does not change in time or at a slow rate so that the hysteresis is negligible:

$$p = \max\left(0, \min\left(1, \frac{R - R_{\min}}{R_{\max} - R_{\min}}\right)\right), \tag{SI 20a}$$

$$\bar{b} = \max\left(0, \min\left(\bar{b}_{R_{\max}}, \frac{R - R_{\min}}{R_{\max} - R_{\min}}\right)\right). \tag{SI 20b}$$

Where $R_{\max} \approx 1.3$ mm/d is the rainfall intensity at $\bar{b}_{R_{\max}} = 12.3$ g/m^2 the average biomass concentration at the point where first gaps emerge in the vegetation cover. $R_{\min} = 0.37$ mm/d is the rainfall intensity at which the last vegetation vanishes. The goodness of fit of the linear approximation of the ground covered by vegetation is $R^2 = 0.97$.

1.7 Modelling the heterogeneity

We model the heterogeneous infiltration coefficient $\mathfrak{a}$ as a geometric stochastic process:

$$\mathfrak{a} = \exp(\mathfrak{z}). \tag{SI 21}$$

This ensures that the coefficients stay positive, as required for both the May and Rietkerk model.

We model $\mathfrak{z}$ as an isotropic stationary stochastic process with first order autocorrelation structure:

$$\mathcal{C}_{\mathfrak{z}}(x, y) = \operatorname{cov}(\mathfrak{z}(x_1, y_1), \mathfrak{z}(x_2, y_2)) = \\ = \frac{\sigma^2}{2\pi\theta^2} \exp\left(-\sqrt{x^2 + y^2}/\theta\right) \quad \text{(SI 22)}$$

Where σ^2/θ is the variance, and θ is the correlation length of $\mathfrak{z}$ where $\mathfrak{z}(0, \theta) = \exp(-1)$. This is a two-dimensional extension of the Ornstein-Uhlenbeck process. Therefore, a is log-normally distributed and with mean and auto-covariance:

$$\bar{\mathfrak{a}} = \exp\left(\bar{\mathfrak{z}} + s_{\mathfrak{z}}^2/2\right), \quad \text{(SI 23a)}$$

$$\mathcal{C}_{\mathfrak{a}}(x, y) = \operatorname{cov}(\mathfrak{a}(0, 0), \mathfrak{a}(x, y)) \\ = \exp\left(2\mu + s_z^2\right) \exp\left(c_{\mathfrak{z}}(x, y) - 1\right). \quad \text{(SI 23b)}$$

An Ornstein-Uhlenbeck process can be interpreted as a first order low-pass filter on white noise. However, we focus in particular on the cases where the characteristic length scale θ is not close to that of the length scale λ_c of the pattern, i.e. we focus on the cases $1/\theta \ll k_c$ and $1/\theta \gg k_c$, which are close to pink and white noise. Note that the limits of pink and white noise themself do not exist, as they do not have integrable densities.

The correlation length $\theta_{\mathfrak{a}}$ of a is:

$$\theta_{\mathfrak{a}} = \theta_{\mathfrak{z}} \log((\log(\exp\left(s_z^2\right) + \exp(1) - 1) - 1)/s_z^2) \leq \theta_{\mathfrak{z}}. \quad \text{(SI 24)}$$

A stochastic process a with desired mean, variance and correlation length can be found by transforming the parameters to that of z accordingly.

1.7.1 Discretization of the heterogeneity

The process is discretized both in the spatial and spectral domain. The discretization truncates the spectral density and autocorrelation, which can result in artefacts similar to aliasing in particular for random fields. We elaborate here on how to reduce these effects.

Discretization in the spatial domain In the numerical model, the process is discretized in space both on a finite spatial domain. For this, we arithmetically average the continuous process over each grid cell:

$$a_{i\,j} = \frac{1}{\Delta x \Delta y} \int_{(i-1/2)\Delta x}^{(i-1/2)\Delta x} \int_{(j-1/2)\Delta y}^{(j-1/2)\Delta y} \mathfrak{a} \, \mathrm{d}x \, \mathrm{d}y \quad \text{(SI 25)}$$

Where $\mathfrak{a}$ is the coefficient of the continuous process and a the discrete model coefficient. This ensures that the spatial structure of the patterns generated with the Rietkerk model are of the grid cell size, as long as the grid cells are sufficiently small compared to the correlation length of the exogenous heterogeneity.

The covariance and correlations differ from that of the continuous process but approach it in the limit $\Delta x \to 0$ and $L \to \infty$.

$$z = \bar{z} + C \circledast e \tag{SI 26}$$

Where C is the covariance matrix of the spatially-averaged process, and e is a random vector with independently standard normally distributed values. This approximates the continuous random field $\mathfrak{a}$ as a discrete random field a of correlated log-normal variables. The values of a are averages of log-normal variables of $\mathfrak{a}$ and hence only log-normally distributed in the limit $\Delta x \to 0$. However, our approach preserves the mean and variance of the distribution and is close to the true process as long as Δx is sufficiently small. The covariance function of the discrete process is found by averaging the covariance function of the continuous process:

$$C_{ij} = \frac{1}{\Delta x \Delta y} \int_{(i-1/2)\Delta x}^{(i-1/2)\Delta} \int_{(i-1/2)\Delta y}^{(i-1/2)\Delta y} \mathcal{C}(x, y)\, \mathrm{d}x\, \mathrm{d}y \tag{SI 27}$$

The discrete, i.e. averaged, processes has a lower variance than the continuous process ($C_{0,0} \leq \mathcal{C}(0,0)$), and higher autocorrelation ($C_{i,j}/C_{0\,0} \geq \mathcal{C}(i\Delta x, j\Delta y)/\mathcal{C}(0,0)$), c.f. Figure 1.7.1. Approximating the integral with a midpoint scheme corresponds to approximating the Ornstein-Uhlenbeck with an first order autoregressive (AR1) process, but this just samples the continuous process at cell centres and hence overestimates the variance and underestimates the correlation at short lag distances. To avoid this, we integrate with a higher order scheme. This corresponds to oversampling in real space.

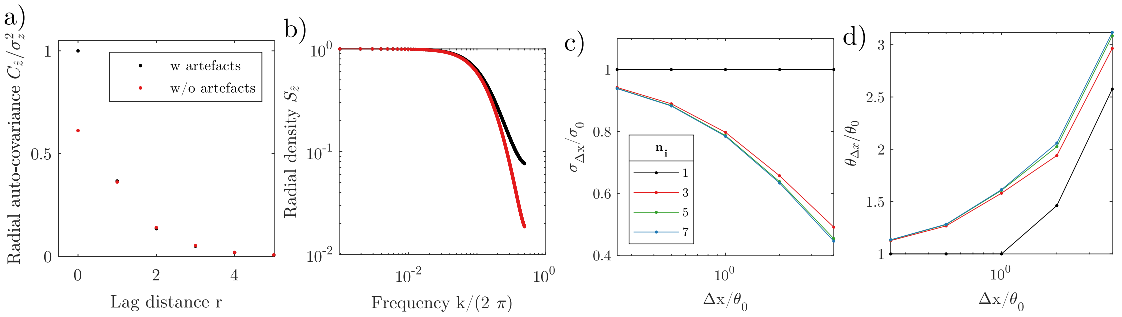

Figure SI 1: Modelling of a c.. by a grid-cell averaging: a) Autocovariance of the discrete geometric Ornstein-Uhlenbeck process, by sampling at cell centres vs by averaging over grid cells. b) Spectral density of the process. c) Decrease of variance with increasing grid cell size, depending on the number of samples per grid cell along one dimension to approximate the average. d) Increase of the correlation length with increasing grid cell size.

Discretization in the spectral domain The finite spatial dimension L can introduce discretization artefacts for processes with long correlation length. Truncation of the autocorrelation to zero outside of the spatial domain results in discontinuous derivatives at the domain boundary, which appear as spurious high frequency oscillation of the spectral density, which appear as spurious vertical and horizontal stripes in the simulated random field. The oscillations increase with the ratio of

the correlation length θ and domain size L. In extreme cases, the oscillation results in an inconsistent spectral density with negative values.

The spurious oscillation can be reduced either by averaging the spectral domain:

$$S_{ij} = \frac{1}{(2\pi)^2} \int_{(i-1/2)\Delta k_x}^{(i+1/2)\Delta k_y} \int_{(j-1/2)\Delta k_y}^{(j+1/2)\Delta k_y} \mathcal{S}(k_x, k_y)\, \mathrm{d}k_x\, \mathrm{d}k_y. \tag{SI 28}$$

We approximate the integral numerically by oversampling spectral density and then averaging by numerical integration. We oversample in frequency space by evaluating the covariance in a domain with a side length that is an integer of the side length of the model domain. Oversampling results in a discrete process with longer correlation length than the continuous process.

The spurious oscillations can also be reduced by applying a smooth Fourier window to the autocorrelation function. Windowing results in a discrete process with shorter correlation length than the continuous process. Note that the two-dimensional discrete Fourier transform is equivalent to the Fourier transform with a rectangular window.

Here, we combine both oversampling and windowing methods to suppress the oscillation to suppress the spurious oscillations with minimal deviation from the correlation length of the continuous process.

2 Parametric linear filters

Stochastic patterns can be generated by filtering uncorrelated noise. Patterns generated by band-pass filtering in particular look strikingly similar to natural isotropic regular patterns, such as spotted, labyrinthine and gapped patterns. The exact expressions for the spectral densities are complicated, even for linearized systems with just two state variables. However, as the linearized systems are already approximations, it is not necessary to determine their density exactly. Instead, we define simple parametric filters, which fit the spectral density reasonably well, and which have parameters that describe the main properties of the spectral properties of the patterns.

The spatial structure of patterns can be analyzed either based on the spectral density $\mathcal{S}$ or based on their autocorrelation function, i.e. characteristic function, $\mathcal{R}$, as they are uniquely related through the Fourier transform. We analyze the densities by decomposing them into two orthogonal components. We decompose the density and autocorrelation of anisotropic patterns into one component $\mathcal{S}_x$, $\mathcal{R}_x$ in the direction perpendicular to the stripes and another component $\mathcal{S}_y$, $\mathcal{R}_y$ in the direction parallel to stripes:

$$\mathcal{S}_{xy}(\vec{k}) = \mathcal{S}_x(k_x) \cdot \mathcal{S}_y(k_y), \tag{SI 29a}$$

$$\mathcal{R}_{xy}(\vec{l}) = \mathcal{R}_x(l_x) \cdot \mathcal{R}_y(l_y). \tag{SI 29b}$$

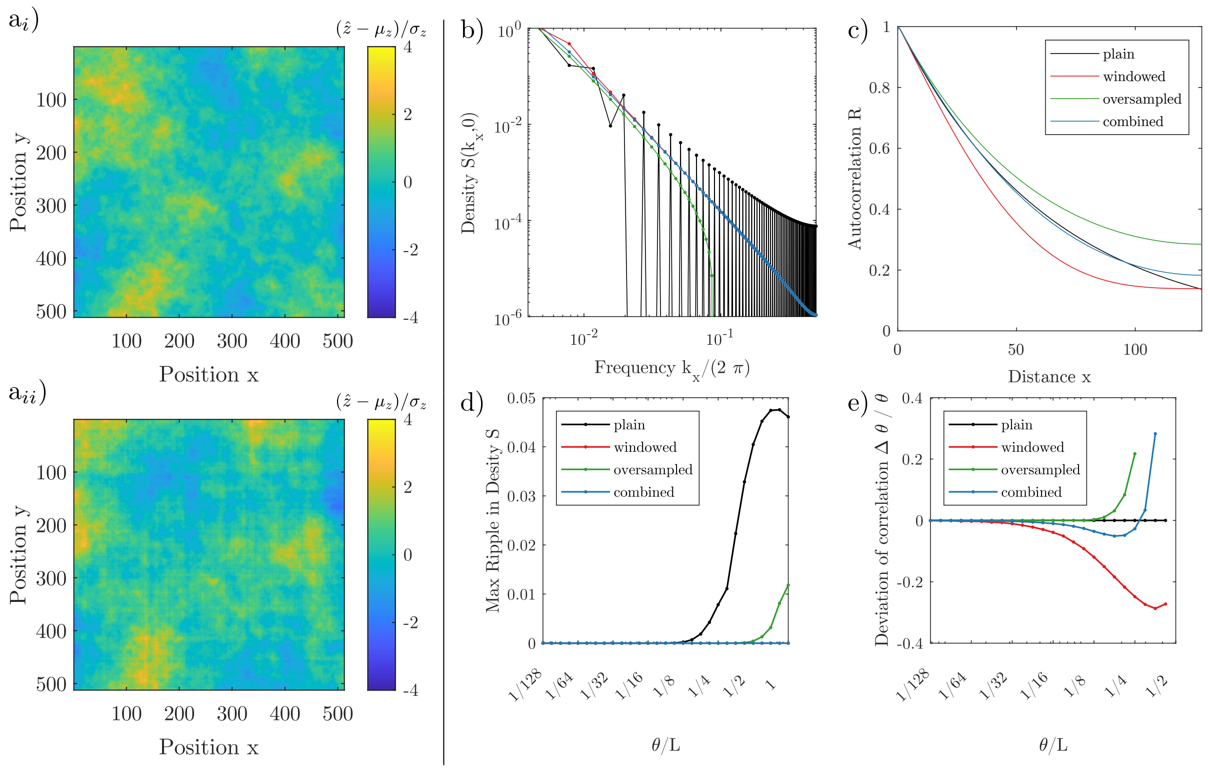

Figure SI 2: a$_i$) Random field with short correlation length ($\theta = 1$) generated by reducing discretization artefacts. a$_{ii}$) Similar random field generated without reducing artefacts. b) Radial autocorrelation and c) spectral density, for several discretization methods. d) Amplitude of the spurious oscillation and e) ratio of the correlation length of the discrete process to that of the continuous process depending on the discretization method.

We decompose the density and autocorrelation of isotropic patterns into a radial and angular component:

$$\mathcal{S}_{r,\theta}(k_r,\theta) = \mathcal{S}_r(k_r)\cdot\mathcal{S}_\theta(\theta), \tag{SI 30a}$$

$$\mathcal{R}_{r,\theta}(r,\theta) = \mathcal{R}_r(r)\cdot\mathcal{R}_\theta(\theta). \tag{SI 30b}$$

Where S_r and R_r are the radial density and autocorrelation, S_θ and R_θ the angular density and autocorrelation, $r = \sqrt{x^2+y^2}$ the radius, $k_r = \sqrt{k_x^2+k_y^2}$ the radial wavenumber, and $\theta = \mathrm{atan}_2\,(k_y,k_x) = \mathrm{atan}_2\,(y,x)$ the angle. The angular density S_θ is usually flat for natural patterns, even if they are regular, and not considered further on.

The analysis of the spectral density is more straightforward than the analysis of the autocorrelation, as the densities of patterns with arbitrary regularity can be modelled by raising a standard density to the p-th power. Taking a higher power corresponds to repeated, i.e. higher order filtering and is associated with higher regular patterns. In contrast, an analysis of the autocorrelation is more cumbersome, as the higher-order filtering corresponds to repeatedly convolving a standard autocorrelation, for which analytic expressions can only be found in simple cases, notably the characteristic functions of the normal and Gamma-distribution mirrored at the origin.

The densities can be expressed as the product of a function with maximum 1, and a factor that normalized the area or or volume to 1, respectively. In general for univariate densities the normalization factors scales as $S_{rc} \propto \lambda_c$ and $S_{xyc} \propto \lambda_c^2$. The ratios S_{rc}/λ_c and $S_{xyc}\lambda_c^2$ are therefore measures of spatial regularity *Kästner et al.* (2024b).

The normalization factor of one-dimensional densities is:

$$S_{xc} = \left(\frac{1}{2\pi}\int_0^\infty \frac{\mathcal{S}_x(k_x)}{S_{xc}}\,\mathrm{d}k_x\right)^{-1}. \tag{SI 31}$$

The coordinate k_x is replaced by k_r when analyzing radial densities, respectively. The reason for not factoring in k_r when analyzing radial densities is that it forces a maximum at 0 and thus prevents us from distinguishing regular from irregular patterns. When analyzing anisotropic patterns, we only have to consider the half-axis, as the spectral density is symmetric, i.e. $S_x(x) = S_x(-x)$. The normalization over the half-axis allows for a direct comparison between the density of a pattern and common univariate densities without having to explicitly mirror them.

The normalization factor of two-dimensional densities is:

$$S_{xyc} = \left(\frac{1}{(2\pi)^2}\int_{-\infty}^\infty\int_{-\infty}^\infty \frac{\mathcal{S}_{xy}(k_x,k_y)}{S_{xyc}}\,\mathrm{d}k_x\,\mathrm{d}k_y\right)^{-1}. \tag{SI 32a}$$

In case of isotropic patterns this is identical to:

$$S_{xyc} = \left(\frac{1}{2\pi}\int_0^\infty k_r\frac{\mathcal{S}_r(k_r)}{S_{rc}}\,\mathrm{d}k_r\right)^{-1}. \tag{SI 33a}$$

In the remainder of the section we derive simple unimodal spectral densities for the analysis of irregular and regular patterns.

2.1 Irregular patterns

We first study the formation of irregular patterns, i.e. patterns which are not self similar. Such patterns typically form in systems without scale-dependent feedbacks. Irregular patterns are typically isotropic. The spatial autocorrelation of irregular patterns decreases monotonically from the origin and therefore has no further local maxima. Correspondingly, the spectral density has its maximum at the origin and no further maxima. The characteristic wavenumber is consequently zero ($k_c = 0$), the characteristic wavelength not finite ($\lambda_c = 2\pi/k_c \to \infty$), and the regularity is zero ($S_{rc}/\lambda_c = 0$).

2.1.1 A nonlinear reaction-diffusion model for irregular patterns

We study irregular patterns with the grazing model by *May* (1977), as detailed in the main manuscript. We run the simulations until $T = 1000[t]$, at which the system is sufficiently stationary $\left(\frac{\partial b}{\partial t} \approx 0\right)$, i.e. hardly changing in time. We choose a computational domain with side length $L_x = L_y = 1000, [L] = [x]$. We discretize the spatial derivatives with the finite difference method and a step size of $\Delta x = \Delta y = 1$. The diffusion part limits the time step of an explicit scheme, like the forward Euler to $\Delta t \leq \frac{\Delta x^2}{4\,e} = 2.5[t]$. Note that our results can be roughly reproduced quickly, i.e. less than 1 sec for low cv(k) and less than 1 min for high cv(k), by reducing the spatial extent to $L = 200$ and end time to $T = 200$.

The system has four critical points: one stable critical point with low biomass, a second stable critical point with high biomass, one unstable critical point in between the high and low biomass, as well as the unstable critical point of bare ground. The system approaches one of two stable critical points depending on whether the initial value is larger or smaller than the unstable critical point separating the plane (Figure SI 3).

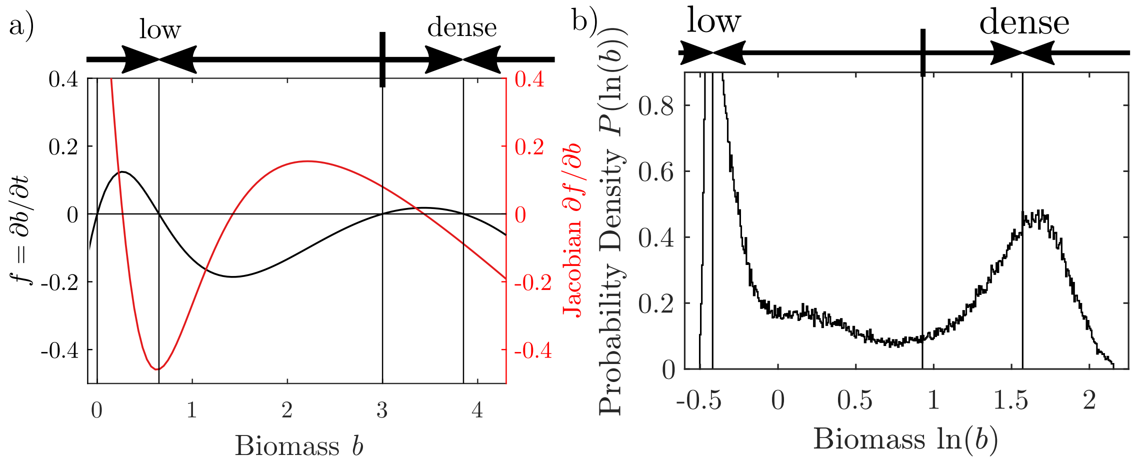

Figure SI 3: a) Trajectory of the non-spatial grazing system, i.e. of the reaction system without diffusion. The system approaches the state of dense vegetation ($b = 0.385[b]$), when its current state is larger than the unstable intermediate state $b = 3.00[b]$, or the state of low vegetation ($b = 0.64[b]$), when it is smaller. b) Probability density of the logarithmic biomass of the spatially extended system. Spatial variation of the carrying capacity k leads to spatial variation of the threshold around 3, and hence to a pattern where the distribution of $b(T)$ is bimodal, i.e. has areas of low as well as of dense vegetation.

In case the system is spatially extended and subject to noise, the system approaches locally different stable states so that a pattern forms. As we do only vary k in space, but not over time, the pattern reaches a stationary state. The degree of heterogeneity $\mathrm{cv}(k)$ does not only influence the spatial structure of a pattern, but also the fraction of ground covered by patches with high biomass. As we only study the spatial structure of the pattern here, we adapt the initial condition b_{ic} for each case depending on the degree of heterogeneity so that patches with high biomass density cover 33% of the ground, Figure SI 4b.

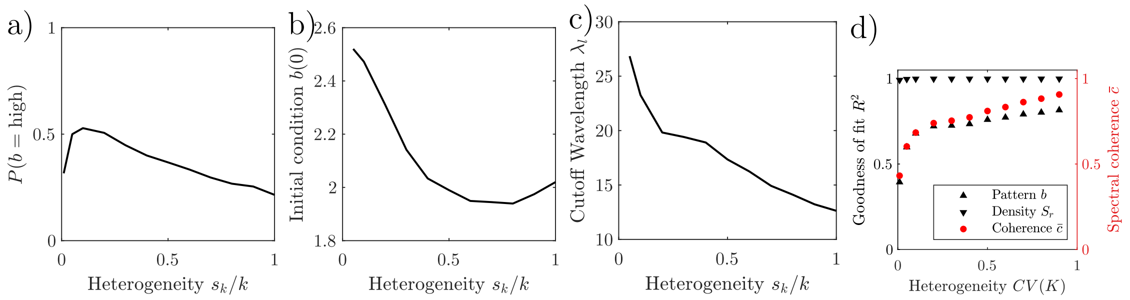

Figure SI 4: a) Fraction of the ground covered with patches of dense vegetation. We keep this fraction constant at 33% by choosing an appropriate b) initial value $b(0)$ depending on the degree of heterogeneity $\mathrm{cv}(k)$. c) Cutoff-Wavelength of the pattern and d) goodness of fit of the pattern generated by low-pass filtering and the spectral density, depending on the degree of spatial heterogeneity $\mathrm{cv}(k)$.

2.1.2 Irregular pattern formation by low-pass filtering

Models generating irregular patterns, like the grazing model *May* (1977), have typically only a single state variable. The linearized operator, c.f. equation SI 9a, is therefore of the form:

$$\frac{\partial \mathcal{A}}{\partial z} = a\,\mathcal{I} + d\,\left(\mathcal{D}_x^2 + \mathcal{D}_y^2\right), \tag{SI 34}$$

where $\mathcal{I}$ is the identity operator. This resembles a first-order low-pass filter. A pattern with the respective spatial structure is generated by filtering white noise e:

$$b_{lp} = (a\,\mathcal{I} - d(\mathcal{D}_x^2 + \mathcal{D}_y^2))^{-1}e. \tag{SI 35a}$$

The transfer function $\mathcal{T}$ is:

$$\mathcal{T}(k_x, k_y) = (a - d\,(k_x^2 + k_y^2))^{-1}. \tag{SI 35b}$$

The spectral density $\mathcal{S}_{xy}$ is:

$$\mathcal{S}_{xy}(k_x, k_y) = \frac{S_{xyc}}{(1 - (k_x^2 + k_y^2)/k_{\mathcal{L}}^2)^2}, \tag{SI 35c}$$

The factors S_{xyc} normalized the volume of the two-dimensional spectral density to 1. The radial spectral density $\mathcal{S}_r$ is:

$$\mathcal{S}_r(k_r) = \frac{S_{rc}}{(1 - k_r^2/k_{\mathcal{L}}^2)^2}. \tag{SI 35d}$$

Where $k_r = \sqrt{k_x^2 + k_y^2}$ is the radial wavenumber and the factor S_{rc} normalizes the area of the radial density to 1. The radial spectral density is equivalent to the spectral density of a low-pass filter in one-dimension.

The spectral density and autocorrelation of the first-order low-pass are uniquely defined by a single parameter, $k_0 = a/d$, which determines the cut-off wavenumber $k_L = k_0\sqrt{2^{1/2} - 1}$ where $S_r(k_{\mathcal{L}}) = \frac{1}{2}S_r(0)$. The filter increasingly suppresses components with higher wavenumber beyond the cut-off.

Arbitrary-order low-pass-filter

Numerical experiments with the grazing model show that the spectrum has indeed the shape of a low-pass filter, but that the magnitude of the high-frequency components decays at a different rate than predicted by the first-order filter. This can be reproduced by a low pass filter with arbitrary order p:

$$b_{lp} = (a\,\mathcal{I} - d(\mathcal{D}_x^2 + \mathcal{D}_y^2))^{-p}e. \tag{SI 36}$$

The filter has the radial spectral density:

$$\mathcal{S}_r = \frac{S_{rc}}{(1 - (k_r/k_c)^2)^{2p}}, \tag{SI 37}$$

with cutoff wavenumber $k_{\mathcal{L}} = k_0\sqrt{2^{1/(2p)} - 1}$.

The constant normalizing the area of the radial density to 1 is:

$$S_{rc} = \frac{2\pi}{k_c}\frac{\Gamma(2p)}{\Gamma(3/2)\Gamma(2p - 1/2)}. \tag{SI 38}$$

Where Γ is the gamma function.

The constant normalizing the volume of the two-dimensional density to 1 is:

$$S_{xyc} = \frac{2\pi}{k_0^2}(2p - 1). \tag{SI 39}$$

The two-dimensional Fourier transform of the spectral density yields the autocorrelation. As the autocorrelation and hence the Fourier transform are radially symmetric, the two-dimensional Fourier transform simplifies into the Bessel transform (*Osgood*, 2019):

$$f = \int_0^\infty r\, R_{LP} \int_0^{2\pi} \exp\left(-i\, r\, k_r \cos\left(\theta\right)\right)\, \mathrm{d}\,\theta\, \mathrm{d}\, r.$$

Where r is the radius, k_r is the radial wavenumber and θ the angle. The spectral density of the low-pass, normalized to unity at the origin, is determined by the radial Fourier transform:

$$\mathcal{S}_{LP,2D}(k_r) = \int_0^\infty r\, R_{LP}(r)\, J_0(k_r\, r)\, \mathrm{d}r \tag{SI 40}$$

Where J_0 is the Bessel function of the first kind. This integral can be solved analytically only for special values of p, but efficiently numerically by applying the projection-slicing theorem and then using the one dimensional Fourier transform. The density of the two-dimensional band-pass filter is then determined by cascading the two-dimensional low-pass filter, as in one dimension. We note that the shape of the radial filters is very close to their one-dimensional counterparts. The cut-off wavenumber of the arbitrary-order low-pass filter is $k_{\mathcal{L}} = k_0\sqrt{2^{1/(2p)} - 1}$, beyond this frequency the radial density decreases asymptotically with a power law with exponent $-4\,p$.
The radially symmetric autocorrelation function is:

$$\mathcal{R}_r(r) = \int_0^\infty k_r\, \mathcal{S}_r(k_r)\, J_0(rk_r)\, \mathrm{d}k_r. \tag{SI 41}$$

Where J_0 is the Bessel function. In the particular case that $p = 3/4$ the autocorrelation function of the two-dimensional low-pass has the shape of a decaying exponential:

$$\mathcal{R}_r(r) = \exp\left(-r/L_{LP}\right), p = 3/4. \tag{SI 42}$$

Where $r = \sqrt{x^2 + y^2}$ is the radial lag distance and The correlation length L_{LP} is the distance at which $R_r = \exp\left(-1\right)$. Both the shape of the autocorrelation function and the correlation length deviate from that of an exponential when $p \neq 3/4$.

2.2 Regular patterns

Regular patterns form in systems with scale dependent feedbacks. Regular patterns are self-similar. Their autocorrelation and spectral density therefore have at least one local maximum distinct from zero. The regularity, measured by the ratio S_{xyc}/λ_c^2, the maximum of the two dimensional density S_{xy}, and the squared wavelength of the maximum, is consequently larger zero. Models of pattern forming systems comprise of coupled reaction-diffusion systems between the feedback takes place. For our simulations, we employ the Rietkerk model, as outlined in the main manuscript. Linear filters can be found as the weighted average of the Rietkerk model linearized at distinct homogeneous states. These filters predict the patterns generated by the full-non linear model well, but finding optimal weights is computationally expensive. We therefore derive simpler filters with fewer parameters for analysis and prediction of patterns.

For isotropic regular patterns, we derive the spectral density of a generalized band-pass filter. Band-pass filtering is realized by low-pass and high-pass filtering in series. High-pass filtering is realized by subtracting the low-pass filtered signal from the unfiltered signal. Repeated filtering results in higher-order filters which increases the selectivity and makes the pattern more regular. A higher order band-pass filter thus can be constructed from a simple low-pass filter.

2.3 Isotropic regular patterns

When fluxes are omnidirectional, i.e. such as for arid vegetation in the plane, the spatial structure of regular patterns, and viz their spectral density is isotropic.

2.3.1 Linear filters for isotropic patterns

For simplicity, we treat here only the case of 2×2 system, as the behaviour of a $k \times k$ system can be approximated reasonably well with a reduced system.
A system is isotropic, when $d_{xi} = d_{yi} = d_i$, and $v_{xi} = v_{yi} = 0$. In particular, when we are only interested in the pattern of one of the state variables, such as the vegetation, and when this state variable is only indirectly sensitive to the perturbation of e, i.e. when $\frac{\partial A}{\partial e} = [0, (\frac{\partial A}{\partial e})_1]$, then the system response is only determined by the block $\left(\left(\frac{\partial A}{\partial z}\right)^{-1}\right)_{12}$:

$$\left(\left(\frac{\partial A}{\partial z}\right)^{-1}\right)_{12} = \left(c_0 + c_1\left(\mathcal{D}_x^2 + \mathcal{D}_y^2\right) + c_2\left(\mathcal{D}_x^2 + \mathcal{D}_y^2\right)^2\right)^{-1} \tag{SI 43}$$

With $c_0 = \det(a)/a_{12}$, $c_1 = (a_{11}d_2 + a_{22}d_1)/a_{12}$, $c_2 = (d_1 d_2)/a_{12}$ and $\det(a) = a_{11}a_{22} - a_{12}a_{21}$. The operator is isotropic with spectral density:

$$\mathcal{S}_r(k_r) = S_I\left(1 - \tilde{c}_1 k_r^2 + c_2/c_1 k_r^4\right)^{-2} \tag{SI 44}$$

As the phase shift is zero, $\mathcal{T} = \sqrt{\mathcal{S}}$.

Where $k_r = \sqrt{k_x^2 + k_y^2}$ is the radial wavenumber and the factor S_I scales the volume

under the density to unity, i.e. $\frac{1}{2\pi}\int_0^\infty k_r\,\mathcal{S}_r\,\mathrm{d}r = 1$.

The spectrum is that of a damped oscillator. The spectral density has a single maximum with wavenumber k_c and density S_{rc}, which determine characteristic wavelength $\lambda_c = 2\pi/k_c$ and the regularity S_c/λ_c of the pattern. The spectral density and therefore the spatial structure, i.e. autocorrelation function, is thus uniquely defined by the two parameters, $\tilde{c}_1$ and $\tilde{c}_2$, which can be uniquely transformed into the characteristic wavelength and regularity.

Four of the six parameters of $\frac{\partial A}{\partial z}$ can be chosen freely when fitting the parameters of $\left(\left(\frac{\partial A}{\partial z}\right)^{-1}\right)_{12}$. A reasonable choice is to choose the diffusion coefficient of the linear system to be identical to the linear system. When the first state variable is directly sensitive to the perturbation, i.e. when $\left(\frac{\partial A}{\partial e}\right)_1$ is not zero, then $\left(\left(\frac{\partial A}{\partial z}\right)^{-1}\right)_{12}$ is also required for determining the pattern. In this case the spectrum of the pattern is slightly more complex and more of the parameters are uniquely determined. Likewise, when approximating the linear operator by a $k \times k$ system, then the shape of the density is similar, but is slightly more complex and influenced by more parameters. However, as the empirical spectral densities of natural patterns are highly uncertain , they can be reasonably well described with a 2×2 system and hence a two-parameter density.

2.3.2 Band-pass filter

Regular patterns form through processes which amplify frequency components around the characteristic wavelength and suppress components that have both lower and higher wavenumbers. This can be reproduced by a band-pass filter. Cascading the low-pass into a high-pass and subsequently band-pass yields:

$$\hat{b}_{BP} = S_c\left(4\left(I - \left(I - \tilde{r}\,(D_x^2 + D_y^2)\right)^{-p_1}\right)^{p_2}\left(I - \tilde{r}\,(D_x^2 + D_y^2)\right)^{-p_3}\right)^{p}\hat{e}, \qquad \text{(SI 45)}$$

The transfer function is correspondingly:

$$\mathcal{T}_{BP} = ((1 - \mathcal{T}_{LP}^{p_1})^{p_2} \cdot \mathcal{T}_{LP}^{p_3})^{p}. \qquad \text{(SI 46)}$$

Where $\cdot$ is the pointwise product. The spectral density is:

$$\mathcal{S}_{BP,r} = S_{BPrc}\left(4\left(1 - \mathcal{S}_{LP}^{\frac{p_1}{2}}\right)^{p_2} \cdot \mathcal{S}_{LP}^{\frac{p_3}{2}}\right)^{2p}. \qquad \text{(SI 47)}$$

Where p_1, p_2, p_3 and p control the shape of the spectral density as well as the regularity of the pattern, and S_c is a scale factor ensuring the conservation of the spectral energy. We choose $p_1 = 1$, $p_2 = 1$ and $p_3 = 1$ as this leads to a simple expression for the spectral density with just one parameter for the regularity. The first-order band-pass behaves similarly to two coupled reaction-diffusion equations or a biharmonic equation which are essential to the formation of patterns in systems with scale-dependent feedbacks.

Cascading the low-pass into a band-pass with the respective values of p_i and normalizing the area to unity yields:

$$S_{BPr} = S_c \left(\frac{2\, k_r\, k_c}{k_r^2 + k_c^2} \right)^{2\,p}. \tag{SI 48}$$

Where p is the filter order, i.e. the number of times the filter is applied in series for the special cases when p is an integer. The filter order determines the selectivity and hence the regularity of the pattern. $k_c = 1/L_{LP}$ is the characteristic wavenumber with corresponding characteristic wavelength $\lambda_c = 2\,\pi\, L_{LP}$. At the characteristic wavenumber, the spectral density reaches the maximum value S_c. S_c also normalizes the area of the spectral density to 1 and is found by integration:

$$S_{BPrc} = \frac{2\,\sqrt{\pi}}{k_c} \frac{\Gamma(p)}{\Gamma(p - 1/2)} \tag{SI 49}$$

Where Γ is the gamma distribution. For the discrete case, i.e. for a finite transect length and sample interval, the relation for the spectral density is slightly more complicated. However, the relation for the continuous case can be fit to sampled natural patterns with negligible deviation.

The factor S_{BPxyc} normalizing the volume of the two-dimensional volume to one is:

$$S_{BPxyc} = \frac{2\pi}{k_c^2} \frac{(2p+1)}{2^{4p-2}} \frac{\Gamma(4p)}{\Gamma(2p+2)\Gamma(2p-1)} \tag{SI 50}$$

2.4 Anisotropic regular patterns

Anisotropic patterns are typically striped and occur in systems where advection is relevant.

2.4.1 Linear filter for regular anisotropic patterns

Anisotropic systems are influenced by advection, i.e. one of the velocities is not zero. The Jacobian contains in general mixed derivatives and is therefore not separable. However, we drop the mixed derivatives to separate the dimensions as this considerably simplifies the expression. With the further simplification that only the second state state variable flows, i.e. v_x is the only nonzero variable, then then $A_i = A_{ix}\, A_{iy}$ with the components:

$$A_{12x} = -\,(a_{12} \det a)(1 + a_{11} v_x(2)/\det a D_x + a_{22}/\det a d_x D_x^2 + d_x v_x(2)/\det a D_x^3)^{-1} \tag{SI 51}$$

$$A_{12y} = -\,(a_{12})((\det a + (a_{11} d_y + a_{22} d_x) D_y^2 + d_x d_y D_y^2))^{-1} \tag{SI 52}$$

The transfer function is:

$$T_x = -\,(a_{12} \det a)(1 + a_{11} v_x(2)/\det a i k - a_{22}/\det a d_x k^2 - i d_x v_x(2)/\det a i k^3)^{-1} \tag{SI 53}$$

$$T_y = -\,(a_{12})(\det a - (a_{11} d_y + a_{22} d_x) k_y^2 + d_x d_y k_y^4)^{-1} \tag{SI 54}$$

And spectral density

$$S_x = S_{Ix}(1 + (c_{x1}^2 - 2c_x2)k^2 + (c_{x2}^2 - 2c_{x1}c_{x3})k^4 + c_{x3}^2 k^6)^{-1} \quad \text{(SI 55)}$$

$$S_y = S_{Iy}(\det a - (a_{11}d_y + a_{22}d_x)k_y^2 + d_x d_y k_y^4)^{-2} \quad \text{(SI 56)}$$

The anisotropic spectrum has two local maxima at $(k_x, k_y) = (\pm k_c, 0)$. Filtering retains x-components near the characteristic wavenumber and y-components near the origin, resulting in stripes parallel to the y-axis. The phase of the x-components is further shifted, i.e. features of the pattern are displaced along the x-direction to perturbations of the parameter, i.e. for vegetation patterns at hillslopes, a perturbation affects the pattern further downhill.

The anisotropic case is considerably more complicated than the isotropic case, as the density has six coefficients. However, we can further simplify the expression to one with just three parameters while still capturing the main features (behaviour near k_c) of the system. The three coefficients determine the characteristic wavelength, the regularity along the x-direction and the cutoff frequency (decay) along the y-direction.

2.4.2 Linear oscillator with phase noise integration

A simpler expression for anisotropic patterns can be found by considering a linear damped oscillator in one dimension:

$$(\mathcal{I} + a_1\,\mathcal{D}_x + a_2\,\mathcal{D}_x^2)b_{LO} = e \quad \text{(SI 57)}$$

Where a_1 and a_2 are coefficients determining the characteristic wavenumber and regularity. The second derivative causes the oscillation, while the first derivative damps it. The filter can be extended in two dimensions by adding a second derivative term in the orthogonal direction. The wavenumber k_c and damping rate r of the oscillation are:

$$k_c^2 = \frac{4\,a_2 - a_1^2}{4\,a_2^2} \quad \text{(SI 58a)}$$

$$r = \frac{a_1}{2\,a_2} \quad \text{(SI 58b)}$$

The damping determines decay of the autocorrelation. The pattern is periodic, when the damping is zero. The pattern is irregular, when the damping is strong ($a_1^2 = 4a_2$, $k_c = 0$). In this case the filter degenerates to a low-pass. Due to the first derivative, the impulse response of the linear oscillator is asymmetric, and the transfer function complex:

$$\mathcal{T}_{LOx} = \frac{2\sqrt{a_1}}{1 + \mathrm{i}a_1\,k - a_2\,k_x^2} \quad \text{(SI 59)}$$

The patterns b can be generated by multiplying with T in the frequency domain. As the filter is linear, b is unimodally distributed around zero, but a bimodal pattern

is obtained after thresholding b. The spectral density of the damped oscillator, and its maximum value at the characteristic wavenumber are:

$$\mathcal{S}_{LOx} = \frac{4a_1}{1 + (a_1^2 - 2a_2)k_x^2 + a_2^2\, k_x^4} \tag{SI 60a}$$

$$S_{LOxc} = \frac{16a_2^2}{4\, a_2\, a_1 - a_1^3} \tag{SI 60b}$$

The density of the linear oscillator is, similar to that of the nonlinear oscillator, not zero near the origin, which sets it apart from the band-pass filter for isotropic patterns. The linear and nonlinear oscillator generate nearly identical patterns for the same noise map ($R^2 \approx 0.98$ for $S_c/\lambda_c = 1$), when phase shifts are ignored and the transfer functions are approximated as $T = \sqrt{S}$. This is because the spectral densities are very close to each other near the characteristic wavenumber k_c, with small differences in the tails.

2.4.3 Nonlinear oscillator with phase noise integration

Anisotropic, i.e. striped, patterns form in reaction-advection-diffusion systems, for example vegetation patterns on hillslopes. The nature of such patterns is well captured by a pattern where the phase is drifting similar to Brownian motion. This model describes stochastic patterns by displacing the stripes: When the coordinate system is rotated so that the stripes run parallel to the y-axis:

$$\frac{1}{\overline{b}_{BM}^2} b_{BM}(x, y) = \sqrt{2}\, \left(1 + \cos\left(k_{BM} x + \varphi(x, y)\right)\right). \tag{SI 61}$$

Where k_{BM} is the wavenumber of the undisplaced pattern. The phase φ is a Brownian surface with covariance:

$$E[\varphi(\vec{x}_1)\varphi(\vec{x}_2)] = \frac{1}{2}(|\vec{x}_1| + |\vec{x}_2| - |\vec{x}_1 - \vec{x}_2|) \tag{SI 62}$$

And $|\vec{x}| = \sqrt{(x/s_x)^2 + (y/s_y)^2}$ the Euclidean distance, with length scales s_x and s_y. The Brownian surface is the generalization of Brownian motion to two dimensions. Along transects, the phase of the pattern undergoes a random walk, and the random phase shift can be interpreted as a random displacement φ/k_{BM}.

Perpendicular to stripes The autocorrelation R_x of the Brownian phase filter in the direction of perpendicular to the stripes is:

$$\mathcal{R}_x = \cos\left(k_c\, l_x\right) \exp\left(-\pi\, k_{BM}\, s_x^2\, |l_x|\right). \tag{SI 63}$$

The Fourier transform of R_x yields the spectral density S_x:

$$\mathcal{S}_x = \frac{1}{k_{BM}} \frac{4\, q\, (q^2 + k_x^2/k_{BM}^2 + 1)}{4\, q^2 + (q^2 + k_x^2/k_{BM}^2 - 1)^2}, \tag{SI 64}$$

with $q = \pi\, s^2$. The area under the density is scaled to 1.

The maximum of the spectral density is located at:

$$k_c = k_{BM}\sqrt{2\sqrt{1+q^2} - q^2 - 1}, \tag{SI 65a}$$

$$S_{xc} = \frac{1}{k_{BM}} \frac{q}{\sqrt{1+q^2} - 1}. \tag{SI 65b}$$

The characteristic wavenumber k_c is slightly larger than k_{BM}. For large degrees of exogenous heterogeneity ($s \geq \sqrt[4]{3}/\sqrt{\pi} \approx 0.74$), the maximum of the spectral density moves to the origin ($k_c = 0$, $S_{xc}/\lambda_c < 0.28$), though it retains an inflection point at 1.86 k_c.

Parallel to stripes The autocovariance in the direction along the stripes is:

$$\mathrm{cov}(b(x,y), b(x, y + l_y) = \int_{-\infty}^{\infty} \cos\left(k_0\, x + \varphi(x,y)\right) \cos\left(k_0\, x + \varphi(x, y + l_y)\right)\, \mathrm{d}y \tag{SI 66}$$

Normalization of the covariance to 1 for $l_y = 0$ yields the autocorrelation:

$$\mathcal{R}_y(l_y) = \exp\left(\frac{-1}{2}\, |l_y|\, s_y^2\right). \tag{SI 67}$$

The spectral density is found via the Fourier transform:

$$\mathcal{S}_y = \frac{4}{\pi} \frac{s_y^2}{s_y^4 + k_y^2}. \tag{SI 68}$$

We have normalized S_y so that the area under the curve integrates to 1. The maximum of the spectral density occurs at 0 and has the value:

$$S_{yc} = \frac{4}{\pi\, s_y^2} \tag{SI 69}$$

The density and therefore the autocorrelation in the direction parallel to the stripes are identical to those of a one-dimensional low-pass filter.

3 Fitting parametric densities and predicting patterns

We fir parametric densities to the one-dimensional components of the two dimensional patterns, i.e. $\bar{S}_x$ and $\bar{S}_y$ for anisotropic patterns, and $\bar{S}_r$ for isotropic patterns. This has two advantages, firstly, it greatly reduces the computational effort, as the components are only one-dimensional, and secondly, the averages $\bar{S}_x$, $\bar{S}_y$ and $\bar{S}_r$ are consistent density estimates, which allows to fit parametric densities directly without smoothing.
We fit the parametric spectral density of a filter to the empirical density of a pattern by minimizing the Hellinger distance. Alternative distances, like the Kullback–Leibler distance perform considerably worse, both with respect to capturing

the mode of the spectral density as well as the visual appearance of the pattern. This is because the KL-distance log-transforms the density, and thus puts heavy weights on the tails. The tails are not very important, as they contain only a small fraction of the spectral energy, and sensitive to noise in satellite images, as image noise is heavy tailed.

The Hellinger distance is the squared difference with respect to the square root of the density:

$$\min_{p} \int_{0}^{\infty} \mathrm{w} \left(\sqrt{\bar{S}} - \sqrt{S_f} \right)^2 \, \mathrm{d}k. \qquad \text{(SI 70)}$$

The Hellinger distance minimizes the error of a pattern generated with the transfer function $T = \sqrt{S}$. Where w is a weight. Here, we choose w in the form of a Gaussian high-pass filter, to suppress spurious low frequency components in natural patterns:

$$\mathrm{w}(k) = \exp\left(-a\, k^2\right). \qquad \text{(SI 71)}$$

We choose the decay rate a, so that w takes the value 0.5 at the wavenumber where the empirical density reaches a minimum between the low frequency lobe centred at 0 and main lobe centred at k_c. We apply the weight both to the parametric density $\tilde{S}$ and empirical density $\bar{S}$, to avoid bias. The weighting can be omitted, when fitting parametric densities to model generated patterns, as those usually do not have spurious low-frequency components.
The steps for fitting the densities are:

1 Compute the periodogram $\hat{S}$.

2 For isotropic patterns:

  a) Estimate the radial density $\bar{S}_r$ from $\hat{S}$.
  b) Fit the parametric density $\tilde{S}_r$ by minimizing the Cramer-von Mises distance.

2 For anisotropic patterns:

  a) Compute the radial periodogram $\hat{S}$.
  b) Compute the angular density from the radial periodogram $\bar{S}_a$.
  c) Determine the principle direction from the maximum of the angular periodogram.
  d) Rotate the periodogram to align the principal direction with the x-axis.
  e) Estimate the densities $\bar{S}_x$ and $\bar{S}_y$ by averaging the rotated periodogram along the respective orthogonal direction.
  f) Fit the density $\tilde{S}_x$ by minimizing the Cramer - von Mises distance.
  g) Fit the density $\tilde{S}_y$ by minimizing the Cramer - von Mises distance.

Figure SI 5: a, d) Regularity S_c/λ_c of patterns generated with the Rietkerk model depending on the degree of exogenous heterogeneity. The higher the degree of exogenous heterogeneity the less regular is the pattern and the flatter is the lobe of the spectral density. The error bars indicate the range in which 50% of values fell for repeated simulations. The vertical lines indicate the value of s_α at which more than 50% of the computer-generated patterns fail the periodicity test at a 5% confidence level. b, e) maximum of the spectral density. c, f) characteristic wavelength.

The repeated filtration with a simple band-pass filter can generate patterns with arbitrary regularity, where the number of repetitions is determined by the selectivity parameter.
Through iterative band-pass filtering, the selectivity of the filter, i.e. the rate at which the density decays away from the central frequency, can be increased. The more selective the filter, the higher and narrower the lobe of the spectral density, and the less rapidly the oscillation of the autocorrelation decays.
The nonlinear phase noise integrator generates true patterns, i.e. spatial structures following a bimodal distribution, with one mode for bare areas and one mode for vegetated areas. This is because the nonlinearity restores the amplitude of the oscillation. However, a two dimensional Brownian surface has to be generated for each pattern, which is computationally expensive (*Stein*, 2002). Visually similar patterns with the same density can be efficiently generated though by approximating the transfer function as $\mathcal{T} = \sqrt{\mathcal{S}}$, or by using a linear oscillator without amplitude restoration.

3.1 Predicting patterns from

We predict patterns with linear filters for isotropic patterns generated by a nonlinear models by filtering the same instantiation of the spatial map e of the heterogeneous model coefficient as used in the nonlinear model:

$$b_{filter} = \mathcal{F}^{-1}(T_{filter} \cdot \mathcal{F}(e)), \tag{SI 72}$$

T_{filter} is the transfer function of a linear filter corresponding to the best fit spectral density S_{filter}:

$$T_{filter} = \sqrt{S_{filter}}. \tag{SI 73}$$

The generic pattern is b_{filter} is then thresholded so that the thresholded pattern covers the same fraction of the ground as the pattern generated with the nonlinear model.

3.2 Goodness of fit of patterns predicted by linear filtration

We estimate the goodness of fit of patterns predicted with the linear filter with respect to those generated with the nonlinear model on hand of the vegetation cover, i.e. after thresholding as:

$$R^2 = (\sin(\pi/2c))^2. \tag{SI 74}$$

Where R^2 is the goodness of fit. Where $c = \mathrm{corr}(b_{l,thresh}, b_{thresh})$ is the correlation of the thresholded patterns. The transformation stems from the equivalent problem of the correlation of signs *Kruskal* (1958). R^2 measures only the goodness of fit of the spatial structure, as the thresholded pattern generated by the nonlinear and linear model have the same mean and variance.

3.3 Random phase

A slight random perturbation of the phase of a periodic pattern yields a pattern with no meaningful information of the phase. We demonstrate this with a nearly-periodic pattern consisting of 8×8 unit meander tiles. We perturb the pattern by slightly displacing it locally by a random distance with low frequency, figure 3.3a. We split the pattern into larger tiles corresponding each to a quadrant of the whole pattern with 4x4 unit meander tiles. As the perturbation is small, the pattern within the quadrants is similar, with a mean pairwise squared correlation of 0.7. The estimated spectral density of the quadrants is nearly identical with a pairwise squared correlation close to 1, figure 3.3c, the phase of the frequency components of the quadrants is not similar and with a pairwise squared correlation close to 0, figure 3.3d. Correlation of the phase angles computed according to *Jammalamadaka and Sarma* (1988).

It might seem surprising at first glance that there is no relation between phase of the quadrants, since the perturbation is small and frequency components of the perturbation are much lower and hence well separated from those of the periodic pattern, but can be understood by the influence of the perturbation on the frequency spectrum: The spectrum of the periodic pattern is identical to that of the unit tiles. It consists of peaks separated by regions where the magnitude of frequency components is zero. The perturbation randomly distributes spectral energy from the peaks to nearby frequency components. The phase of the new components depends on the perturbation and is therefore not well defined, i.e. a random value between 0 and 2 π. In contrast, the expected magnitude of the new components depends on the spectral density of the stochastic process of perturbation and that of the unit tile, and it is well defined, i.e. can be revealed by smoothing the periodogram. The redistributes of the spectral energy in presence of a small low frequent displacement follows from the nature of the Fourier transform and trigonometric identities.

References

Jammalamadaka, S. R., and Y. R. Sarma, A correlation coefficient for angular variables, *Statistical theory and data analysis II*, pp. 349–364, 1988.

Johnston, N., and D. Greene, *Conway's Game of Life: Mathematics and Construction*, Nathaniel Johnston, 2022.

Kästner, K., R. C. van de Vijsel, D. Caviedes-Voullième, N. T. Frechen, and C. Hinz, Unravelling the spatial structure of regular dryland vegetation patterns, *Catena*, *247*, 2024a.

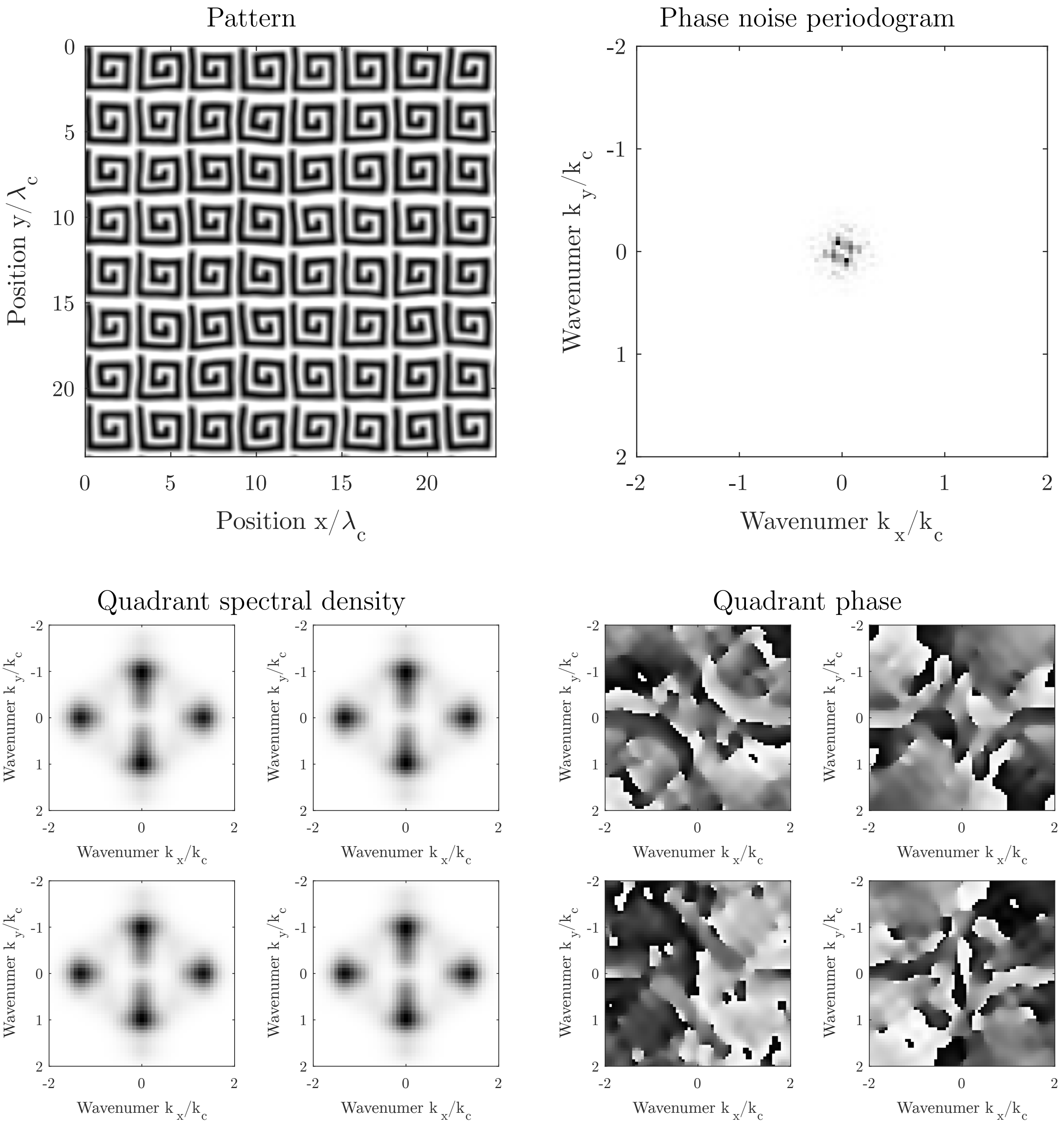

Figure SI 6: a) Highly regular pattern generated by slightly perturbing the phase of a periodic pattern with low-frequent noise b) Periodogram of the phase noise c) Spectral density of each quadrant, estimated by smoothing the periodogram d) Smoothed phase of each quadrant. Determined from smoothing the sine and cosine of the phase. Jumps at quadrants boundaries have been smoothed by multiplying with a Tukey window.

Kästner, K., R. C. van de Vijsel, D. Caviedes-Voullième, and C. Hinz, A scale-invariant method for quantifying the regularity of environmental spatial patterns, *Ecological Complexity*, *60*, 2024b.

Kruskal, W. H., Ordinal measures of association, *JASA*, *53*(284), 814–861, 1958.

May, R. M., Thresholds and breakpoints in ecosystems with a multiplicity of stable states, *Nature*, *269*(5628), 471–477, 1977.

Osgood, B. G., *Lectures on the Fourier Transform and its Applications*, vol. 33, American Mathematical Soc., 2019.

Rietkerk, M., M. C. Boerlijst, F. van Langevelde, R. Hille Ris Lambers, J. van de Koppel, L. Kumar, H. H. Prins, and A. M. de Roos, Self-organization of vegetation in arid ecosystems, *The American Naturalist*, *160*(4), 524–530, 2002.

Stein, M. L., Fast and exact simulation of fractional Brownian surfaces, *Journal of Computational and Graphical Statistics*, *11*(3), 587–599, 2002.
