# Supplementary material for: Formation of spatial vegetation patterns in heterogeneous environments
Source: PLoS One. 2025 May 28;20(5):e0324181. doi: 10.1371/journal.pone.0324181 (PMC12118857; doi:10.1371/journal.pone.0324181)
Supplement: Attachment — Submitted filename: review-1-response-pone-2025-01-15.pdf [file pone.0324181.s002.pdf]

Response to review of the revision of: Spatial pattern formation in heterogeneous environments
PONE-D-24-45670

Karl Kästner et al.

January 15, 2025

Contents

Editor's comments **1**

1 Reviewer #1 **2**

2 Reviewer #2 **6**

3 Reviewer #3 **8**

Our response to comments is written in blue. Line numbers in our response refer to the revised manuscript, if not otherwise indicated.

Editor's comments

Dear Dr. Kästner,

Thank you for submitting your manuscript to PLOS ONE. After careful consideration, we feel that it has merit but does not fully meet PLOS ONE's publication criteria as it currently stands. Therefore, we invite you to submit a revised version of the manuscript that addresses the points raised during the review process.

[. . .]

Kind regards,

Pan Li, PhD
Academic Editor
PLOS ONE

Dear Editor Pan Li,

Thank you for sending us the reviews of our manuscript and encouraging us to submit a revision. We addressed the issues raised by all three reviewers.

Considering the concerns of the first reviewer, we have backed our conceptual ideas with a more

formal analysis in section 2. We have also restructured and streamlined the manuscript to make it easier to follow.

We hope that you find our improvements to your liking and will gladly make further adjustments if requested from your side.

Kind regards,

Karl Kastner

Reviewers' comments:

1 Reviewer #1

1. Is the manuscript technically sound, and do the data support the conclusions?
Reviewer #1: Partly

2. Has the statistical analysis been performed appropriately and rigorously?
Reviewer #1: I Don't Know

3. Have the authors made all data underlying the findings in their manuscript fully available?
Reviewer #1: Yes

4. Is the manuscript presented in an intelligible fashion and written in standard English?
Reviewer #1: Yes

5. Review Comments to the Author

The authors explore the formation of irregular and regular (but not perfect) vegetation patterns, suggesting that their formation resembles the actuation of a filter on top of heterogeneities.

The manuscript is not easy to follow,

We thank the first reviewer for his critical comments. We thoroughly revised the manuscript to make it easier to follow.

and the relevance of the findings is not clear to me.

Our manuscript offers a novel perspective on regular pattern formation, c.f. abstract, sentence 8. We want to inspire the community which perceives regular pattern formation solely as a deterministic process.

The authors claim that vegetation patterns in nature are not perfectly periodic as the ones formed by models, and I agree with this. However, it is not direct to claim that a linear filter over an external heterogeneity is all that it takes to reproduce them. The dynamics of such a macroscopic system (vegetation cover) are highly nonlinear and hardly mimicked by a linear process.

We do not claim that the pattern formation is linear. We interpret pattern forming systems, and reaction-advection-diffusion models thereof, as a nonlinear filters, c.f. line 90. While this is only a new moniker, it comes along with a novel mindset, recognizing the influence of random exogenous spatial heterogeneity, and pattern formation as a stochastic process.

It should also be kept in mind that a model is not necessarily comprehensive just because it is nonlinear. Common nonlinear reaction-advection-diffusion (RAD) models extremely simplify the complex biophysical processes in ecosystems. Approximating the response to noise by linearization is therefore not as drastic as it seems, c.f. line 726. We find that linear filters approximate the spatial structure of natural patterns reasonably well, i.e. they explain the full spectral density and autocorrelation. Linear filters explain half the spatial variance of a pattern forming in heterogeneous environment, c.f. line 504, which is considerably better than nonlinear model with idealized homogeneous environments, c.f. line 728 and *Kästner et al.* (2024a,b). We also discuss differences between patterns generated by linear filters and the nonlinear filters, c.f. section 7.2.

Furthermore, it is known that linear processes either diverge or collapse to zero, making them unsuitable to describe the formation of patterns.

This is a misconception, we extended section 2 to demonstrate this. In short, consider the nonlinear reaction diffusion system of the form:

$$\frac{\partial z}{\partial t} = A(z, e). \tag{1}$$

Expand the series and only keep the constant and linear terms:

$$\frac{\partial z_l}{\partial t} = A(z_*, e_*) + \frac{\partial A}{\partial z}(z - z_*) + \frac{\partial A}{\partial e}(e - e_*) \tag{2}$$

z_l approaches the stationary state $z_{l,\infty}$, when the eigenvalues of $\frac{\partial A}{\partial z}$ are negative:

$$z_{l,\infty} = z_* - \left(\frac{\partial A}{\partial z}\right)^{-1} \left(A(z_*, e_*) + \frac{\partial A}{\partial e}(e - e_*)\right) \tag{3}$$

Collecting the terms $\tilde{z}_* = z_* - \frac{\partial A}{\partial z}^{-1} A(z_*, e_*)$ and $L^{-1} = \frac{\partial A}{\partial z}^{-1}\frac{\partial A}{\partial e}$

$$z_{l,\infty} = \tilde{z}_* - L^{-1}(e - e_*). \tag{4}$$

When z_* and e_* are spatially homogeneous, then $\tilde{z}_*$ is spatially homogeneous and L is space invariant so that all the spatial variation in $z_{l,\infty}$ originates from e, i.e. the pattern generated by the linear system is a linear transformation of the exogenous heterogeneity.

Additionally, it is not clear to me whether the external heterogeneity is fixed on time or time-dependent,

The heterogeneity in our examples is only spatial, i.e. constant in time line 302. Though our results carry over to spatio-temporal noise.

as the authors claim that they impose a mask on the nonlinear models,

We are not sure what the reviewer is referring to as "applying a mask to nonlinear models". Can

the reviewer further elaborate on their comment so that we can provide a meaningful answer with the next revision?

but also mention that constant perturbations will balance with filtering, finally reaching an equilibrium. Again, I disagree with this, as a linear process does not reach equilibria as mentioned above.

See our reply to the comment above.

The authors mention that in an isotropic environment, the phase in the Fourier transform has no effect. I think this is not true, as the phase dictates what pattern one will observe. Take for example a labyrinth and a disordered hexagonal pattern of the same wavelength, they will show a similar power spectrum, but the phase is what determines their morphology (labyrinthine patterns have a much more disordered phase).

The reviewer is correct that the nonlinear processes influence the phase. We discuss this in section 7.2. This is the main reason why linear filtering explains only about half the variance, c.f. line 504. However, a linear model can still reproduce the spatial structure of natural regular patterns better than nonlinear models with idealized homogeneous environments, as the former predicts at least the spectral density and therefore the autocorrelation, while the latter does not, line 728.

Spotted, gapped and labyrinthic patterns can furthermore be distinguished without considering the phase. In fact the three pattern types emerge after thresholding the generic pattern generated by a linear filter at different levels, c.f. line 239. The main effect of the nonlinearity is that it determines the fraction of the ground covered by vegetation, i.e. the area covered by the patches, while the linearized system predicts the spatial arrangement of the patches rather well.

I found it weird that the authors declare contributions for four authors, but only three are listed on the manuscript. The same happens on the supplementary material, which shows four authors, in discrepancy with the authors listed in the manuscript.

We thank the reviewer for spotting this. We had a contributor in the early phase of the project who unfortunately could not contribute to this manuscript due to his limited availability.

This supplementary material is also hard to read, with incomplete phrases that abruptly end, such as the one at the end of section 2 of the supp. Material.

We revised the supplementary material making it more comprehensible.

Overall, I feel that the methods are not clearly explained,

We hope that our response and additions to the manuscript and supplement clarify our methodology.

and the conclusions, such as "...we further demonstrate that patterns form through filtering of the noise..." require more study to support them, as again, those systems are highly nonlinear.

We agree that the topic is worth further research, though we strongly believe that our evidence is strong enough and our results insightful enough to be published.

This is readily seen by the authors when they themselves increase the 'noise intensity', seeing worse matches between the nonlinear models and the linear filters.

Important is that linear filtering generates patterns which are similar to natural regular patterns for intermediate degrees of heterogeneity, in particular since nonlinear models assuming homogeneous environments do not. We also note that the goodness of fit depends slightly on the initial condition and correlation structure of the noise. In the supplement we provide an example where the initial vegetation cover is uniform, in this case the goodness of fit increases up to 0.8 for higher degrees of heterogeneity, c.f. Figure SI 3.

For these reasons, I can not recommend the article for publication.

6. PLOS authors have the option to publish the peer review history of their article (what does this mean?). If published, this will include your full peer review and any attached files.
Reviewer #1: No

2 Reviewer #2

1. Is the manuscript technically sound, and do the data support the conclusions?
Reviewer #2: Yes

2. Has the statistical analysis been performed appropriately and rigorously?
Reviewer #2: Yes

3. Have the authors made all data underlying the findings in their manuscript fully available?
Reviewer #2: Yes

4. Is the manuscript presented in an intelligible fashion and written in standard English?
Reviewer #2: No

The authors investigated a novel understanding of spatial pattern formation in ecosystems by incorporating stochastic processes and scale-dependent feedbacks and highlighted how these patterns can be influenced by various factors, such as noise and scale-dependent biophysical feedbacks. However, there are some issues that need to be addressed carefully. The comments can be found below. I recommend revisions.

Comment 1. Natural ecosystems are rarely isolated. External influences, boundary effects, and environmental gradients (e.g., climate or topography) may significantly influence pattern formation but appear underemphasized in the framework.

While these questions are interesting, the manuscript does not have enough space to elaborate on them in detail. Inhomogeneous boundaries are just a spatial heterogeneity which is limited to the boundaries and are therefore indirectly covered by our theory. However, interesting phenomena occur due to the finite spatial extent. We refer to several studies where the influence of boundaries has been studied (, 9, 0, 1, 2), c.f. line 694.

Exogenous heterogeneities influence the spatial structure qualitatively similar for different water availability, i.e. climate. This is because the climate primarily determines the total area of the patches and only slightly affects the spatial arrangement of the individual patches, c.f. line 785.

In addition, temporal dynamics are also crucial in understanding ecosystem functioning. The role of temporal changes in pattern formation is not explicitly addressed in the article. It is recommended that these issues [are] briefly discussed as potential directions for future works.

We discuss some of our observations with that respect: With respect to temporal changes we find that the spatial structure of the patterns are considerably less history dependent than patterns generated in idealized homogeneous environments. We also find that the spatial structure, i.e. the autocorrelation and spectral density, do not strongly depend on the water availability, which might indicate that the spatial structure is not a suitable indicator of ecosystem health, line 795 We also find that the random exogenous heterogeneity slow the uphill migration of banded patterns, c.f. line 564.

Comment 2. The whole text needs to be checked in terms of typos and punctuation errors.

We thoroughly revised and proofread the text.

6. PLOS authors have the option to publish the peer review history of their article. [...] If you choose "no", your identity will remain anonymous but your review may still be made public.
Reviewer #2: No

3 Reviewer #3

1. Is the manuscript technically sound, and do the data support the conclusions?
Reviewer #3: Yes

2. Has the statistical analysis been performed appropriately and rigorously?
Reviewer #3: N/A

3. Have the authors made all data underlying the findings in their manuscript fully available?
Reviewer #3: Yes

4. Is the manuscript presented in an intelligible fashion and written in standard English?
Reviewer #3: Yes

The study by Kästner et al. deals with the formation of spatial patterns in the biomass density of some species (e.g. vegetation in arid systems) in the presence of environmental heterogeneity. The authors claim that models that explain pattern formation through self-organization but ignore environmental heterogeneity predict idealized patterns that are far more regular than the patterns observed in nature and that by taking environmental heterogeneity into account, both regular and irregular patterns can be predicted that resemble their natural counterparts more closely. The most important result the authors present is that in heterogeneous environments the process of pattern formation can be understood as a filtering of the random spatial heterogeneities. In case of regular patterns, scale-dependent feedbacks act as a band-pass that amplify components of the noise (i.e., the random environmental heterogeneities) corresponding to the system's characteristic wavelength while suppressing all other components (on both shorter and longer spatial scales). In case of irregular spatial patterns, diffusion is shown to act as a low-pass that filters out short-scale components of the noise.

Overall, I consider this manuscript to be an interesting and important contribution to the literature on spatial pattern formation in ecological systems. It is very well written and meets, as far as I can tell, all publication criteria of PLoS ONE. I have a number of rather minor comments the authors may find helpful to further improve the presentation of their work.

We thank the reviewer for his encouraging words and detailed helpful comments.

1) The introduction is very accessible and provides a good overview of the relevant literature. To further improve it, the authors might provide an intuitive description of what "filtering" means in the context of their study. Since this is such a central concept in this study (and it certainly becomes clear later on), readers not familiar with it might benefit from some verbal explanations early on.

Explanation one line 90.

2) In the paragraph following line 81 it could be mentioned that the positive short-range feedback in vegetation models is due to improved water infiltration by established vegetation. In line 185 the authors mention infiltration without ever having stated its significance.
Explained on line 43.

3) line 106: this looks like an incorrect figure reference. I guess the authors mean Fig. 1b_ii,c_ii.

Corrected.

In general, panels of figures are not labeled consistently. Often, letters (a, b, c) with additional numerals (i, ii, iii) are used (e.g. Fig. 1), sometimes only letters (e.g. Fig. 3), and in Fig. 5 even a mix of both styles. At times, even the authors get confused by this (see e.g. line 383 or line 395). I recommend to use one style consistently.

We made the indexing consistent, but kept the individual scheme, where we label related panels belonging to the same numerical experiment or pattern with the same letter and Roman index, and unrelated panels with individual letters.

4) Figure 1, caption: typos in the second line (a_i instead of a_ii) and at the very end (unnecessary 'et al.'). Also, I noticed that in this figure, 'autocorrelation' was used for the iii)-panels, whereas later (e.g. Fig. 3) 'correlogram' was used. Sure, the terms mean the same thing, but why not make it easier for the readers and use one term consistently?

Corrected "radial densities" to "correlograms" in Figure 3. Our draft figures included radially averages and we had missed to update this caption. Corrected "autocorrelation" to "correlogram" in Figure 1.

Note that we distinguish the correlogram (= sample autocorrelation of a single pattern) from the autocorrelation of the system or model, in the same way we distinguish the periodogram (= spectrum of a single pattern) from the spectral density of a system or model. We explain this in the revised manuscript in the caption of Figure 1.

5) line 211 and Fig. 2: it is never clearly stated how the 'mirror image' is defined.

We avoid the wording in the revised text and figure to avoid confusion. Note that convolving b with its mirror image $b_{mirror}(x, y)) = b(-x, -y)$ yields the autocorrelation, as, after subtracting the mean and normalizing the pattern by its variance, then the autocorrelation is $R(\tau) = E[b(x)b(x+\tau)]$, while the convolution is as $(b \circledast b)(\tau) = E[b(x)b(x - \tau)]$.

6) lines 270-274: It looks like a few things got confused here. First, the sentence starting 'The characteristic wavenumber...' should probably start with 'In anisotropic patterns, the characteristic wavenumber...' to clearly separate it from the text preceding it (which is about isotropic patterns). The following sentence should be adjusted accordingly. Also, the authors first use 'x' for the direction perpendicular to the stripes (line 271/272) and immediately thereafter 'x' is the direction parallel to the stripes and 'y' is perpendicular to the stripes (line 273/274).

Corrected.

7) First paragraph of Section 3.1: While I agree with the authors that keeping most of the mathematics out of the main text improves it readability and accessibility, I still think that providing the main equations of the May and Rietkerk models (i.e., Equations 1 and 5da-5de in the Appendix) in the main text would be helpful. The authors frequently refer to parameters of these models, which is a bit awkward if the equations are not shown.

We state the model equations in the revised manuscript in section .

8) line 326: typo: 'are and are' →'and are'
Corrected.

9) Figure 5: the caption mentions a dashed horizontal line in panel a), but the line appears to be missing in the panel.

Line added.

10) line 562: again, I recommend to use technical terms consistently throughout the manuscript. Here, 'spectral energy' is used for the first time, before the authors used 'spectral density'.

We rephrased the respective sentences avoiding the term spectral energy. Note that we define the spectral energy as the (not normalized) part of the variance contributed by a frequency component, while the spectral density is the (normalized) fraction of the variance contributed by a frequency component, i.e. spectral energy is the product of the variance (a scalar) and the spectral density (a function with unit volume).

11) lines 619 and 649, Fig. 9 and 10: same thing, only here the authors use the term 'bands' to describe anisotropic patterns, everywhere else they use 'stripes'. Please unify!

Corrected. We used vegetation bands in the draft manuscript, but changed to stripes to avoid confusion with the term bandpass.

12) Figure 10, panel f: I think the title should be 'Perpendicular to bands' (or rather: ... stripes) and the label on the y-axis should be 'Autocorrelation R_x'.

We mistakenly repeated the subplot of S_y instead of plotting R_x. We replaced the subplot against the correct one.

13) line 694: include a reference to Fig. 7b_i here, as there patterns generated with the Rietkerk model are shown. Also, the statement of the following sentence ('The spectral density ...') is not supported by Fig. 1c_iii alone. To which results should the readers compare this?

The revised manuscript displays the density fit in Figure 12.

14) The numbering of the equations in the Appendix needs to be fixed.

We added the prefix SI to all references within and to the supplement.

6. PLOS authors have the option to publish the peer review history of their article. [...] If you choose "no", your identity will remain anonymous but your review may still be made public.
Reviewer #3: No
