# Supplementary material for: Formation of spatial vegetation patterns in heterogeneous environments
Source: PLoS One. 2025 May 28;20(5):e0324181. doi: 10.1371/journal.pone.0324181 (PMC12118857; doi:10.1371/journal.pone.0324181)
Supplement: Attachment — Submitted filename: pone-review-2-response-2025-03-30.pdf [file pone.0324181.s003.pdf]

Response to review of the revision of: Formation of spatial vegetation patterns in heterogeneous environments PONE-D-24-45670R1

Karl Kästner et al.

March 30, 2025

Contents

Editor's comments **1**

1 Reviewer #1 **2**

2 Reviewer #2 **5**

Our response to comments is written in blue. Line numbers in our response refer to the revised manuscript, if not otherwise indicated.

Dear Dr. Kästner,

Thank you for submitting your manuscript to PLOS ONE. [...] we invite you to submit a revised version of the manuscript that addresses the points raised during the review process. We look forward to receiving your revised manuscript.

Kind regards,

Pan Li, PhD
Academic Editor
PLOS ONE

Dear Editor Pan Li,

Thank you for sending us the review of our revised manuscript. We addressed all comments by the reviewer who remained sceptical. In particular, we demonstrate that our findings apply to all kinds of regular patterns, including spotted, labyrinthine and gapped patterns with additional simulations. We also demonstrate that isotropic patterns can be reasonably well predicted by linear filters with an additional analysis of the cross-spectra between the patterns and the exogenous heterogeneity.

We hope that you find our revision satisfactory.

Kind regards,
Karl Kastner

1 Reviewer #1

1. If the authors have adequately addressed your comments raised in a previous round of review and you feel that this manuscript is now acceptable for publication, you may indicate that here to bypass the "Comments to the Author" section.
Reviewer #1: (No Response)

2. Is the manuscript technically sound, and do the data support the conclusions?
Reviewer #1: Partly

3. Has the statistical analysis been performed appropriately and rigorously?
Reviewer #1: I Don't Know

4. Have the authors made all data underlying the findings in their manuscript fully available?
Reviewer #1: Yes

5. Is the manuscript presented in an intelligible fashion and written in standard English?
Reviewer #1: Yes

6. Review Comments to the Author
Reviewer #1:

7. PLOS authors have the option to publish the peer review history of their article (what does this mean?). If published, this will include your full peer review and any attached files.
Reviewer #1: No

6. Review Comments to the Author
Reviewer #1: I thank the authors for the revised version. The manuscript is generally in a much better shape, being readable and understandable. Now that I better understand the objective of the authors, I must highlight the following issues I have with the manuscript:

a) I think there is a confusion throughout the manuscript when referring to the effect of the heterogeneity as stochastic. While it is true that the authors employ heterogeneity 'masks' generated by a random process (this is what I mean by mask, the function describing the heterogeneous parameter), this does not render the dynamic stochastic. The dynamical system remains deterministic, as there is no fluctuating in time forcing. In other words, the system is not a stochastic differential equation, it remains a partial differential equation (or ordinary differential equation when space is discretized). This should be made clear, as various other works study the effect of stochasticity in its own, which is a problem tackled with different techniques.

   Differential equations are referred to as stochastic when their coefficients randomly vary either in time or *space* (*van Kampen*, 1976; *Vanmarcke*, 1983; *Holden et al.*, 1993; *Botte et al.*, 2023). Every pattern is unique, but the spectral density and spatial correlation structure of the patterns are the same when they for in an environment with statistically identical conditions, c.f. line 89. The spectral density of the stochastic process is the probability density of how the spatial variance is distributed over the frequency component of a pattern.

   This also carries over to the techniques. We adjust the (co)-variance of the randomly varying

coefficient with respect to the grid cell area in a similar manner as as the variance of temporal noise is adjusted with respect to the time-step (*Vanmarcke*, 1983; *Botte et al.*, 2023), c.f. line 422.

We also relate striped patterns to a noisy oscillator in one spatial dimension, c.f. section . As the equations of the one-dimensional noisy oscillator are the same irrespectively if the oscillation takes pace in space or time, it is meaningful to refer to both cases as stochastic and treat them with the same methods.

b) The explanation of the linearization of the system is still not satisfactory to me. I must highlight that the eigenvalues of the Jacobian ($\partial A / \partial z$) do not need to be negative if you are linearizing around an arbitrary state. In particular, the case of pattern-forming systems makes this approach ambiguous. The authors state that they linearize around a homogeneous state. In a pattern forming system in the pattern forming regime, this will lead to eigenvalues both negative (for long and short wavelength perturbations) and positive (for perturbations around the Turing wavelength). Then, employing initial conditions with a non-negative projection in the unstable directions of the phase space will make the linear system diverge (even in the presence of spatial heterogeneities). The authors must acknowledge this limitation, and should be aware that their approach is correct only for linearizations very close to the equilibria of the nonlinear system (in the example I elaborated, near a patterned state and not a homogeneous state).

The reviewer is correct that the homogeneous state is unstable as otherwise no pattern would form. However, we focus on the spatial structure of the stabled patterned state, which the system approaches over time. At this point the system is stable c.f. line 150 and line 250. We show that it is always possible to find a stable homogeneous linear operator reproducing the atuocorrelation structure of the pattern, c.f. line 165.

c) My previous point highlights why I stressed so much the importance of nonlinearities. Linearizing around a homogeneous state in a pattern-forming system will render equation (4) confusing. It indeed predicts an equilibrium, but it may be an unstable one. Thus, the authors might be reporting unstable states throughout the manuscript. The authors must take special care when explaining this. This problem is avoided when applying the method to a non-pattern forming system. There, linearizing around a homogeneous state ensures the whole spectrum of eigenvalues is negative.

It is correct that nonlinearities are important for stability. Though we are interested in the spatial structure, i.e. the autocorrelation function of the patterned state, not the stability of homogeneous states line 147. We also determine the homogenous linear operator by stochastic linearization, not by linearizing at a single (unstable) homogeneous state, line 165.

There is a study where researchers analyze the effect of spatial heterogeneities in the parameters on a non-pattern forming model and find that heterogeneities explain many of the features exhibited in real vegetation covers, such as the power spectrum, the correlations, and even the patch size distribution [*] [*]: Vegetation covers phase separation in inhomogeneous environments, Chaos, Pinto-Ramos et a., Solitons & Fractals 163, 112518 (2022)

Pinto-Ramos et al 2022 had not yet been published when our current manuscript was conceived. Pinto-Ramos conducted very similar experiments as *van Nes and Scheffer* (2005) which we

referenced. Pinto-Ramos employs a reaction-diffusion model with cubic reaction term. While the model employed by P-R is nonlinear, it is even simpler than the Rietkerk model, and can hardly be considered an holistic representation of the complex nonlinear processes in plants and soils.

The mathematical analysis in Pinto-Ramos is more thorough than that in *van Nes and Scheffer* (2005). However, the spectral analysis of Pinto-Ramos is inconsistent. They assess that the irregular patterns in their study have a spectrum that resembles a power law and an autocorrelation that resembles a decaying exponential. This is a contradiction in itself, as the spectrum and autocorrelation are Fourier transform pairs, and it is well known that the Fourier transform of a power law is a power law, not an exponential (*Gradshteyn and Ryzhik*, 2014). The Fourier transform of an exponential has a similar shape to the density of a low-pass filter, c.f. line 454.

d) I remain curious about the authors' choice to neglect the phase of the Fourier transform of patterns.

We do not neglect the phase. However, the phase shift of the linear part of an isotropic filter is always zero, c.f. line 217, while the phase of the patterns is effectively random. The phase of the components predicted by the linear filter, which explain most the spatial variance, is therefore identical to the phase of the environmental heterogeneity, and thus random and its analysis not insightful, c.f. line 220. The frequency components generated by the nonlinearity causes a.o. the bimodality of the biomass distribution, c.f. line 288. However, while the nonlinearity restores wavelength and amplitude, it does not restore the phase line 770. Components introduced by the nonlinearity are coupled, though the randomness of the heterogeneity propagates to the nonlinear components. The slight perturbation of the phase of a periodic pattern for example results in a spectrum without any order in the phase, see the experiment provided in the supplement, section 3.3. However, the random deviation of the phase with respect to that of a periodic pattern forming in a noise-free oscillator determines the spatial correlation structure and hence regularity of a pattern. We derive the relation between phase deviation and spatial structure for the case of a simple oscillator, c.f. section . The lack of meaningful information in the phase is a common property of nonlinear noisy oscillators, which are therefore analyzed based on their spectral density, i.e. the magnitude of the frequency response alone *Demir et al.* (2000). We note that the *Echeverría-Alar et al.* (2023) referred to by the reviewer neither investigate the phase, likely for the same reason.

- My main issue is regarding labyrinthine patterns, which are ubiquitous in vegetation cover patterns. They also fall inside the category of isotropic patterns, but are much more complex than hexagonal ones. The authors must discuss how their method applies to labyrinthine patterns. Maybe the authors will find relevant a study where the role of spatial heterogeneities in the parameters is studied in the labyrinthine patter-forming regime of vegetation cover models [**]. There, it is shown how changing the intensity of heterogeneities and their correlation length (they have a spatial structure) affects the power spectrum of such patterns, showing that they can be ideal, irregular, or scale-free. This highlights that heterogeneous parameters in nonlinear models are highly relevant to understand vegetation cover patterns. [**]: Effect of heterogeneous environmental conditions on labyrinthine vegetation patterns, Echeverría-Alar et al, Physical Review E 107 (5), 054219 (2023)

  We demonstrate that our theory applies to regular patterns irrespectively if they are spotted,

labyrinthine or gapped with additional model runs, c.f. section . Spotted, gapped and labyrinthine patterns differ primarily in the mean biomass and less so in their spatial correlation structure, c.f. section . This is a consequence of the definition of the autocorrelation which is independent of the mean, c.f. line 584.

Echeverría-Alar 2023 was published after the main of our manuscript was written, we refer to it in the revised supplement, c.f. line 78. t is encouraging that Echeverría-Alar et al obtained similar results to ours. Our interpretation is wider, we realize that the effect of a heterogeneous environments is similar for spotted, gapped and labyrinthine patterns. We also identify the pattern formation as a stochastic process, and derive representative spectral densities, albeit for simplified processes. Echeverría-Alar et al. in contrast merely fit ad-hoc a Lorentzian density without relating it to the process et all.

e) In line with my points regarding the importance of nonlinearities in the study of vegetation patterns, recent studies have shown how including heterogeneous parameters in models of vegetation cover modifies the diagram of states, that is, the vegetation pattern morphology as a function of driving parameters such as the aridity. There, researchers show that irregular and regular patterns could be alternative stable states induced by heterogeneity [***], highlighting the relevance of nonlinearities. Then, authors must be careful when presenting their work, as filtering could be a useful idea to understand the phenomenon, but only (very) close to already known equilibrium states. [***]: Vegetation clustering and self-organization in inhomogeneous environments, arXiv preprint arXiv:2406.12581 (2024)

   We agree that nonlinearties and heterogeneity can interact to increase the range in which stable patterns form. Yet, we are interested in the spatial correlation structure of the patterns, not in the stability of homogeneous states.

- Overall, I think the manuscript has merit and could make a relevant piece of work for the community. However, the authors must acknowledge the weaknesses of their approach more carefully and not deem the nonlinear nature of these complex systems as a burden, as it can be highly relevant even when the current models used to study it are just qualitative or phenomenological. For these reasons, I can not recommend to publish this manuscript.

  We thank the first reviewer for their comments which helped making the manuscript more understandable to a general audience. We think that our evidence is fairly convincing: the spectral coherence shows that frequency components are to a large degree predictable by a linear filter, patches in patterns generated with the Rietkerk model align with a sinusoidal heterogeneity when the frequency of the heterogeneity is close to the characteristic length scale of the system, and finally, patterns predicted by linear filter fit well the patterns generated by the Rietkerk model when the exogenous heterogeneity is random.

7. PLOS authors have the option to publish the peer review history of their article. [...] If you choose “no”, your identity will remain anonymous but your review may still be made public.
Reviewer #1: No

2 Reviewer #2

1. If the authors have adequately addressed your comments raised in a previous round of review and you feel that this manuscript is now acceptable for publication, you may indicate that here.

Reviewer #2: All comments have been addressed

2. Is the manuscript technically sound, and do the data support the conclusions?
Reviewer #2: Yes

3. Has the statistical analysis been performed appropriately and rigorously?
Reviewer #2: Yes

4. Have the authors made all data underlying the findings in their manuscript fully available?
Reviewer #2: Yes

5. Is the manuscript presented in an intelligible fashion and written in standard English? Reviewer #2: Yes

6. Review Comments to the Author Reviewer #2: (No Response)

7. PLOS authors have the option to publish the peer review history of their article. [...] If you choose "no", your identity will remain anonymous but your review may still be made public.
Reviewer #2: No

We thank the second reviewer for their evaluation.

References

Botte, W., E. Vereecken, and R. Caspeele, Random field modelling of spatial variability in concrete–a review, *Structure and Infrastructure Engineering*, pp. 1–14, 2023.

Demir, A., A. Mehrotra, and J. Roychowdhury, Phase noise in oscillators: A unifying theory and numerical methods for characterization, *IEEE Transactions on Circuits and Systems I: Fundamental Theory and Applications*, *47*(5), 655–674, 2000.

Echeverría-Alar, S., D. Pinto-Ramos, M. Tlidi, and M. Clerc, Effect of heterogeneous environmental conditions on labyrinthine vegetation patterns, *Physical Review E*, *107*(5), 054,219, 2023.

Gradshteyn, I. S., and I. M. Ryzhik, *Table of integrals, series, and products*, Academic press, 2014.

Holden, H., T. Lindstrøm, B. Øksendal, J. Ubøe, and T.-S. Zhang, Stochastic boundary value problems: A white noise functional approach, *Probability theory and related fields*, *95*, 391–419, 1993.

van Kampen, N. G., Stochastic differential equations, *Physics reports*, *24*(3), 171–228, 1976.

van Nes, E. H., and M. Scheffer, Implications of spatial heterogeneity for catastrophic regime shifts in ecosystems, *Ecology*, *86*(7), 1797–1807, 2005.

Vanmarcke, E., *Random Fields: Analysis and Synthesis*, MIT Press, 1983.
